# Genome-Wide Characterization and Expression Analysis of GeBP Family Genes in Soybean

**DOI:** 10.3390/plants11141848

**Published:** 2022-07-14

**Authors:** Sushuang Liu, Yanmin Liu, Chundong Liu, Feixue Zhang, Jiaping Wei, Bingxuan Li

**Affiliations:** 1 Department of Life Sciences and Health, Huzhou College, Huzhou 313000, China; liu@zjhzu.edu.cn (S.L.); liuyanmin@zjhzu.edu.cn (Y.L.); liuchundong@zjhzu.edu.cn (C.L.); 2 Institute of Crop, Huzhou Academy of Agricultural Sciences, Huzhou 313000, China; feixue66666@126.com; 3 Gansu Province Key Laboratory of Aridland Crop Sciences, Lanzhou 730070, China; weijp@gsau.edu.cn; 4 The Key Laboratory for Quality Improvement of Agricultural Products of Zhejiang Province, College of Advanced Agricultural Sciences, Zhejiang A&F University, Hangzhou 311300, China

**Keywords:** soybean (*Glycine max* L.), *GeBP* gene family, genome-wide analysis, trichome

## Abstract

The glabrous-enhancer-binding protein (GeBP) family is a family of plant-specific transcription factors, whose members share a central DNA-binding domain. Previous studies have already proven that *GeBP* genes are involved in the control of cell expansion but not cell proliferation in *Arabidopsis*. However, there has not yet been a versatile analysis of the *GeBP* genes’ function in soybean (*Glycine max* L.). Here, we identified and named 9 *GmGeBP* genes in the soybean genome. These genes were distributed on 7 of the 20 chromosomes and the intron numbers ranged from zero to one. According to the phylogenetic tree, 52 *GeBP* genes obtained from four plant species were clustered into major four groups. Through the RNA-seq analysis of the nine *GmGeBP* genes, 8 of 9 *GmGeBP* genes were be found to expressed differentially across the 14 tissues. Additionally, among nine *GmGeBP* genes, only *GeBP4* were highly expressed in abnormal trichome soybeans, which was predicted to be involved in trichome development. This genome-wide analysis of *GmGeBP* genes helps to provide an overview of the evolution and functions of two kinds of soybean plants. These results will help to clarify the potential functions and characteristics of *GmGeBP* genes in the soybean life cycle.

## 1. Introduction

The soybean is regarded as an important oil and food crop with a high protein content around the world, and it provides a vital source of human food, animal feed and cooking oil [1]. However, soybean plants are often exposed to various harmful environments, such as salinity, drought, pH and temperature variations and heavy metals, which limit soybean production. In order to alleviate the damage caused by environmental conditions, the plants developed rich protective structures during evolution [2]. For example, the plants’ epidermal hair, which is a special kind of skin structure, is widely distributed amongst land plants and protects the plants from environmental stress [3,4].

Transcription factors (TFs) are important regulators of developmental processes and stress response. In plants, TFs often bind the cis-acting element to affect gene expression in response to stress, eventually protecting against or reducing damage to plants. The glabrous-enhancer-binding protein (GeBP) family is a family of transcription factors specific to plants, whose members share a central DNA-binding domain [3,5]. In Arabidopsis, *GALBROUS1* (*GL1*) was an early gene discovered to regulate the initiation of epidermal hair growth, the glabrous-enhancer-binding protein can regulate the occurrence of epidermal hair by adjusting the expression pattern of *GL1* [6]. The conserved motif analysis of the Arabidopsis GeBP sequence revealed that it possesses a basic amino acid region and a leucine zipper region, suggesting that GeBP may belong to the bZIP transcription factor family. However, it was found that the interval between these two conserved domains exceeds 9 residues, which is not a standard definition of a bZIP protein [7,8]. N-x7-R/K-x9-L-x6-L-x6-L is the basic structure of a typical bZIP conserved domain, which includes alkaline domains and Leu zipper domain separated by 9 residues. Among the 21 GeBP family members in Arabidopsis, the research has focused on the similarities of the GeBP/GeBP-LIKE formation of a unique clade and the sharing of an undefined extra-endian conserved region [3].

It is worth noting that the *GEBP/GPL* gene represents a newly defined class of leucine zipper (Leu zipper) transcription factors, and they play redundant roles in the regulation of the cytokinin hormone pathway [9]. A recent study demonstrated the Arabidopsis GeBP-LIKE 4 (GPL4) transcription factor as an inhibitor of root growth that is induced rapidly in the root tips in response to cadmium (Cd) [10]. These research outcomes suggested that the *GeBP* family gene is not only involved in the developmental process of the plant but also protects against environmental stress. The constitutive expressor of pathogenesis-related gene-5 (*CPR5*) in Arabidopsis (*Arabidopsis thaliana*) displayed highly pleiotropic functions, particularly in pathogen responses, cell proliferation, cell expansion and cell death. It was found that GeBP/GPLs are involved in the control of cell expansion in a CPR5-dependent manner but not in the control of cell proliferation by regulating a set of genes that represents a subset of the CPR5 pathway [11].

The soybean is an ancient tetraploid, and is the main oil and protein crop around the world. Although *GEBP* gene members in Arabidopsis and rice have been characterized [3], a comprehensive analysis of *GmGeBP* at the genome-wide level in soybean is still needed. Hence, we identified and characterized the *GmGeBP* gene family in soybean at the genome-wide level. The cis elements of the *GmGeBP* promoter region were also analyzed. The expression patterns of *GmGeBP* genes in several tissues and under different treatments such as drought, salinity, cold and heat were investigated through the publicly available transcriptome data and our quantitative real-time polymerase chain reaction (qRT-PCR) results. Our study will not only widen the gene information on the *GmGeBP* family in soybean, but will also provide a useful source and new insights for scientists to analyze the functions of *GmGeBP* in the future.

## 2. Results

### 2.1. Identification of GeBP Gene Family in Soybean

A total of 9 members of the *GmGeBP* gene family were distributed on 7 of 20 soybean chromosomes, while *GeBP3* and *GeBP4* existed on Chromosome 10, *GeBP8* and *GeBP9* existed on chromosome 20, respectively (Table 1). The bioinformatics analysis of 9 members showed that they had different characteristics in terms of their amino acid length and WM and pI values. The amino acid lengths ranged from 353 to 448, the molecular weights ranged from 39.647 to 49.235 kDa and the pI values ranged from 4.65 to 9.08 (Table 1). No significant differences were found between the acids and bases among the GeBP proteins, except one (*GmGeBP2*), which was basic. Wolf PSORT prediction was used to determine the subcellular locations of all GmGeBP proteins. Among the proteins, 8 members of the GmGeBP proteins most likely localized in the nucleus, while the GmGeBP8 protein localized in the cytoplasm.

### 2.2. Chromosomal Distribution and Intron–Exon Patterns of GmGeBP Genes

**Figure 1:**
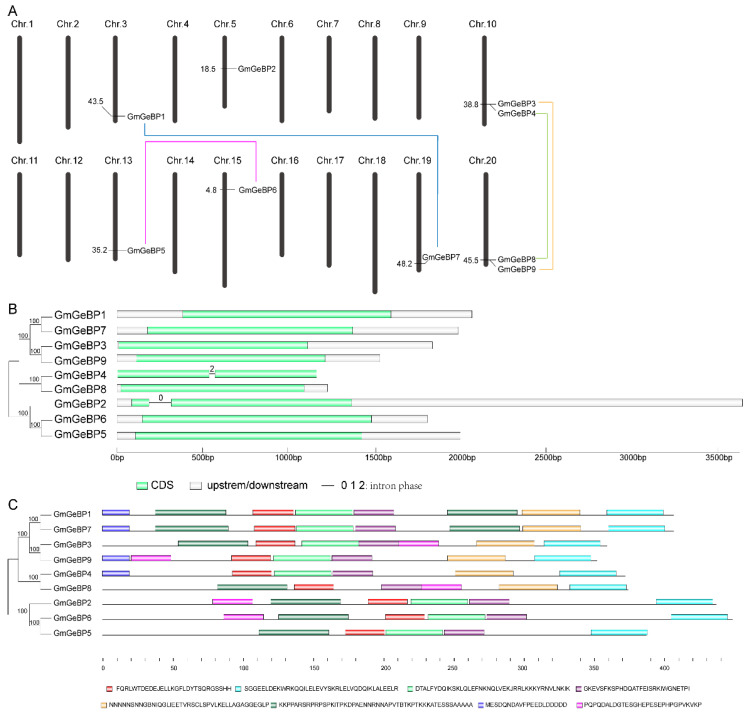
Characteristics of *GmGeBP* genes. (**A**) Chromosomal map and gene duplication events of *GmGeBPs*. Only the locations of *GeBP* genes are shown in the map. The segment duplication events are marked by different colored dotted lines. (**B**) Gene structures of *GmGeBP* genes. The unrooted phylogenetic tree consists of 9 *GmGeBP* genes. The different colored boxes represent different types of motifs. (**C**). Structures of conserved motifs of GmGeBP proteins. The unrooted tree was produced using the MEGA version 11.0 program using the amino acid sequences of all nine GmGeBP members with the neighbor-joining (NJ) method. The boxes in different colors represent the different motifs (the boxes in the first line from left to right represent motifs 1, 2, 3 and 4 and the boxes in the next line from left to right represent motifs 5, 6, 7 and 8) and the name of each motif is displayed on the right of the box. The length of a protein and motif can be measured using the scale at the bottom.

As shown in Figure 1A, the physical positions of the *GmGeBP* genes were obtained from the Phytozome database, and were used to map them to their corresponding chromosomes. Seven out of the twenty chromosomes possessed *GmGeBP* genes. The number of *GmGeBP* genes on each chromosome was only one or two. In detail, chromosomes 10 and 20 harbored two *GmGeBP* genes, while each of the remaining five chromosomes (chromosomes 3, 5, 13, 15 and 19) contained only one *GmGeBP* gene. Additionally, the location of each *GeBP* family member on the soybean chromosome was marked in the picture and the detailed data are shown in Table 1. The gene duplication events were also analyzed and the segmental duplication patterns are connected by blue lines in Figure 1.

The gene structure divergence occupies an important place in the evolution of gene families and provides extra evidence for the analysis of phylogenetic relationships [12]. Therefore, the intron–exon configurations of *GmGeBP* genes were constructed using the Gene Structure Display Server (Figure 1B). The results obtained from the analysis of the gene structure of *GmGeBP* family members showed that the number of introns among the genes equaled only one. Only two *GmGeBP* genes (*GmGeBP2* and *GmGeBP4*) were among the *GeBP* gene family members that had one intron and were clustered similarly. The remaining genes had no intron. It was also suggested that the structure of the *GmGeBP* genes was relatively stable and was not prone to variable shearing when replicating. The CDS sequence data and genomic sequence data are shown in Supplementary Files S1 and S2.

### 2.3. Conserved Motif Analysis of GmGeBP Proteins

The analysis provided the three conserved motif patterns of GmGeBP proteins obtained from MEME (Figure 1C). The number of motifs that each GmGeBP protein contained was changed from five to eight. GmGeBP1 and GmGeBP7 contained eight conserved motifs and GmGeBP3 and GmGeBP9 contained seven conserved motifs. Additionally, half of the GmGeBP proteins (GmGeBP4, GmGeBP8, GmGeBP2 and GmGeBP5) contained six conserved motifs, while GmGeBP6 only had five conserved motifs. Motifs 1, 2, 3 and 4 were widely presented in all of the GmGeBP proteins, which indicated that they were conserved and might play important roles. Motif 5 existed in the proteins of GmGeBP7 1, 3, 4, 7, 8 and 9, which could be deemed to be one clade. Motif 6 in GmGeBP1 and GmGeBP7 proteins was shown in two locations. Furthermore, motif 7 was presented at the beginning of the GmGeBP1, GmGeBP7, GmGeBP4 and GmGeBP9 proteins. In general, closely related GmGeBP proteins on adjoining branches of the phylogenetic tree had the same or a similar motif constituent. For example, GmGeBP1 and GmGeBP7 both had eight conserved motifs. The low sequence diversity of GmGeBP protein domains suggested that the *GmGeBP* family members were stable after the genome duplication.

### 2.4. Phylogenetic Analysis of GmGeBP Genes in Plants

**Figure 2:**
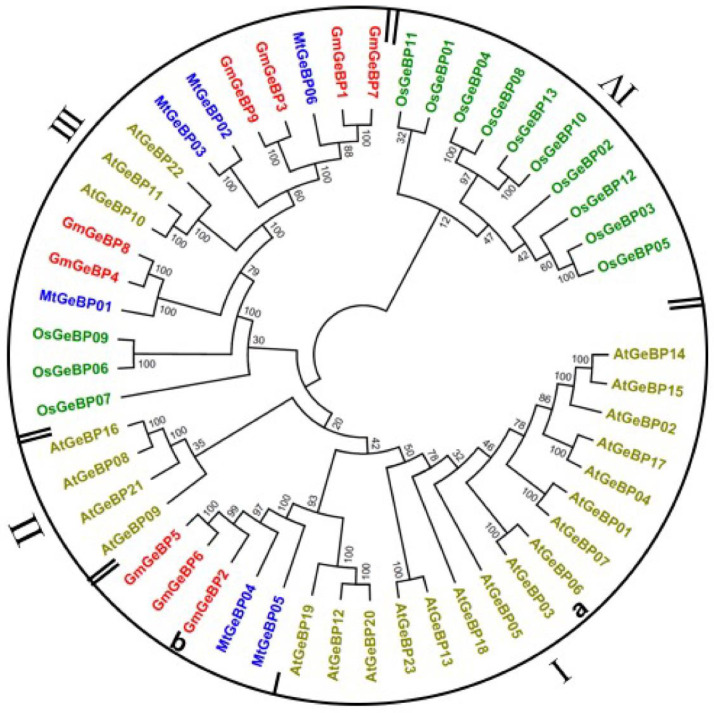
The phylogenetic tree of GeBP proteins from soybean and other plants, including *Arabidopsis thaliana*, *O. sativa* and *Medicago sativa* L. The phylogenetic tree was constructed using MEGA version 11.0 with the neighbor-joining (NJ) method. The members of each GeBP gene family are indicated with the same color.

To investigate the phylogenetic relationships and the evolutionary history of GeBP proteins in soybean, a total of 52 *GeBP* proteins from *Arabidopsis thaliana*, *Glycine max*, *Oryza sativa* and *Medicago sativa* L. were used to construct an unrooted phylogenetic tree with the neighbor-joining method. As shown in Figure 2, the *GeBP* members from different plant species were divided into four major groups named I, II, III and IV. Group I was the largest subfamily, which contained 21 members, while group II was the smallest with four *OsGeBP* members. In detail, group I could be subdivided into two subgroups (a and b) according to the bootstrap values. The phylogenetic analysis showed that the *GmGeBP5*, *GmGeBP6*, *GmGeBP2* and *MtGeBP* genes clustered in subgroup b. As depicted in group III, which had the majority of the *GmGeBP* gene members, *GmGeBP4* grouped with *GmGeBP8*, *GmGeBP3* grouped with *GmGeBP9* and *GmGeBP1* grouped with *GmGeBP7* were clustered in this clade. As can be seen from the whole picture, it is interesting to note that the *GmGeBP* genes had a closer relationship with lucerne than the other species among them. Most GmGeBP proteins in the same group shared identical motifs and similar exon–intron patterns among the related genes. This was consistent with the fact that soybean and lucerne are both legumes.

### 2.5. Analysis of Cis-Elements in Putative GmGeBP Gene Promoters

**Figure 3:**
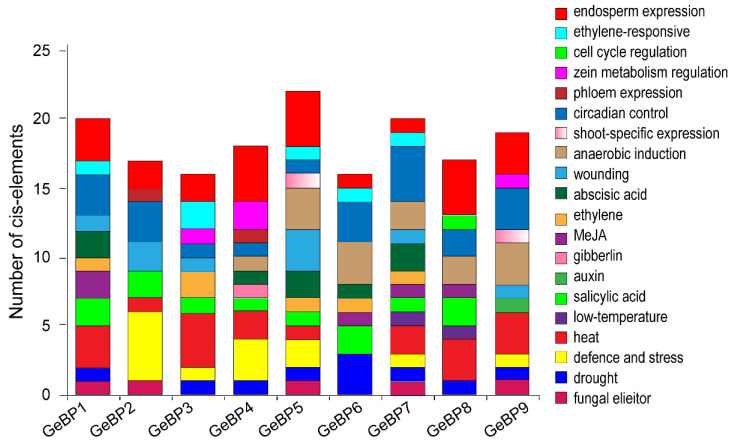
Cis-elements in the promoters of putative GmGeBP genes. Different cis-elements with the same or similar functions are present with the same color.

To investigate the regulation patterns of *GmGeBP*, the cis-elements of the *GmGeBP* gene promoter were analyzed. The 2000 bp sequence upstream of the start codon of each *GmGeBP* gene was determined using Phytozome software. The cis-elements were divided into seven categories: stress response, hormone response, light response, promoter-related, development-related, site-binding-related and unknown. Elven stress-responsive cis-elements were identified, including *HSE*, *LTR*, *box S*, *TC-rich repeats*, *W box*, *WUN-motif*, *MBS*, *MBSI*, *MBSII*, *GC-motif* and *Box-W1*, which reflected plant responses to heat, low-temperature, defense stresses, drought, anaerobic induction and fungal elicitors, respectively. Six kinds of hormone-responsive cis-elements were certified, including salicylic acid, MeJA, gibberellins, auxin and ethylene (Figure 3). Nine kinds of development-related cis elements were identified, such as *as-2-box*, *circadian*, *AC-I*, *AC-II*, *O2-site*, *MSA-like*, *ERE*, *GCN4_motif* and *Skn-1_motif*, which influenced the shoot-specific expression, circadian control, phloem expression, zein metabolism regulation, cell cycle regulation, ethylene induced expression and endosperm expression. A relatively large number of light-responsive cis-elements, promoter-related cis-elements and site-binding-related cis-elements in *GmGeBP* promoters were observed (Supplementary Files S5 and S6).

### 2.6. RNAseq Analysis of GmGeBP Genes in Various Tissues

**Figure 4:**
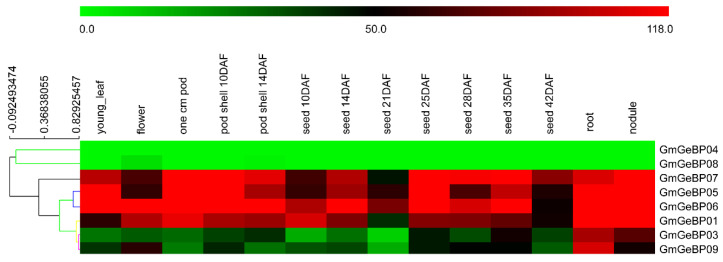
Expression heat map of GmGeBP genes in 14 tissues. RNA-seq relative expression data from 14 tissues were assessed to obtain the expression patterns of soybean genes. The raw data were normalized and retrieved from Soybase.

To further investigate the expression patterns of *GmGeBP* genes during soybean development, the RNA-seq atlas data were used to analyze the expression profiles of 14 different tissues (young leaf, flower, one cm pod, pod shell 10 DAF, pod shell 14 DAF, seed 10 DAF, seed 14 DAF, seed 21 DAF, seed 25 DAF, seed 28 DAF, seed 35 DAF, seed 42 DAF, root and nodule) in the soybean cultivar William 82. The RNA-Seq atlas data of *GmGeBP* genes were downloaded from Soybase (http://soybase.org/soyseq/, accessed on 23 October 2021) [13]. However, the RNA-Seq atlas data of *GmGeBP2* were not obtained, which might indicate that this gene is a pseudogene or is only expressed at specific developmental stages or under special conditions. As shown in Figure 4, we observed that the eight *GmGeBP* genes were mainly clustered into two classes on the hierarchical clustering analysis.

Class I contained two *GmGeBP* members (*GmGeBP4* and *GmGeBP8*) that were not expressed in these fourteen tissues. Class II contained six *GmGeBP* gene members (*GmGeBP1, 3, 5–7, 9*), and all of the *GmGeBP* genes in this class were obviously upregulated in all fourteen tissue types. Here, two *GmGeBP* gene members (*GmGeBP3* and *GmGeBP9*) revealed expression differences compared with other members in this class. At the young leaf stage to the flower stage, two genes (*GmGeBP5 and GmGeBP7*) were found to be downregulated, while the other six *GmGeBP* genes revealed upregulation. Half of the genes exhibited transcript abundance profiles with marked peaks in the tissues from the one cm pod, and four (*GmGeBP1, 3, 5, 7*) *GmGeBP* gene members were downregulated at two stages of the pod–shell phase. At the seed stage, the expression patterns of four (*GmGeBP3, 5, 6, 7*) *GmGeBP* gene members increased at 14 DAF, 25 DAF and 35 DAF but fell at 21 DAF, 28 DAF and 42 DAF. Meanwhile, the other 2 genes’ expression profiles dropped at 14 DAF, 25 DAF and 35 DAF but increased again at 21 DAF, 28 DAF and 42 DAF. It was found that the majority of genes except two (*GmGeBP3* and *GmGeBP9*) in this class examined were significantly expressed in the tissues of the roots and nodules.

### 2.7. Expression Analysis of Soybean GeBP Genes in Response to Trichome Development

**Figure 5:**
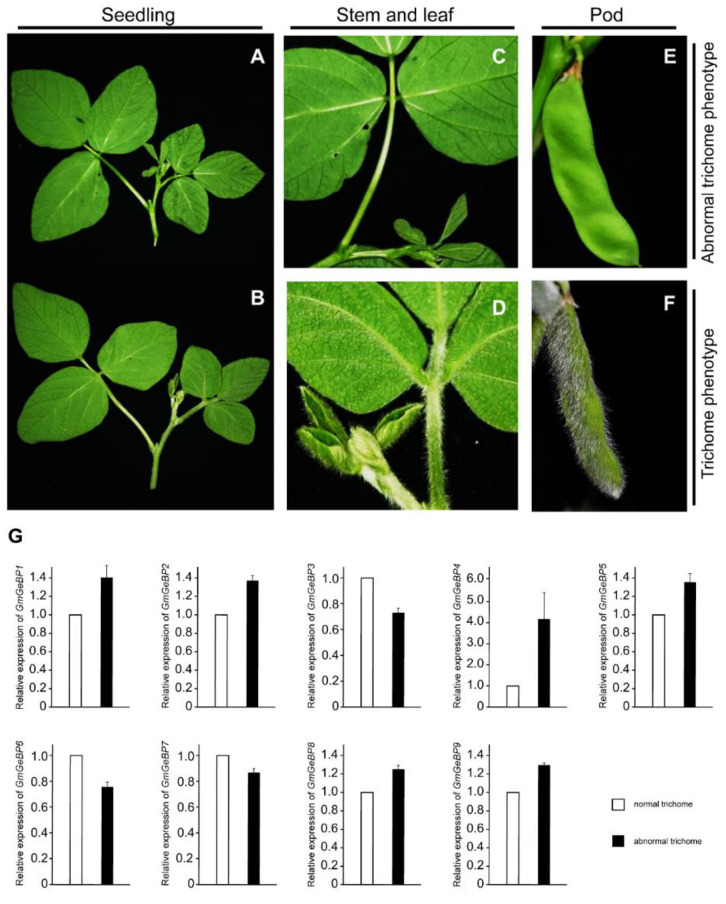
Expression levels of *GmGeBP* genes in abnormal and normal trichome soybean. (**A**,**C**) Phenotype of abnormal trichome soybean. (**B**,**D**) Phenotype of trichome soybean. (**E**) Phenotype of abnormal trichome soybean pod. (**F**) Phenotype of trichome soybean pod. (**G**) Quantitative RT-PCR was used to investigate the expression levels of GmGeBP genes, and the results are represented by means ± standard deviations. The relative expression levels of *GmGeBP* genes in abnormal trichome soybean are compared with those in trichome soybean.

In order to analyze the expression profiles of *GmGeBP* genes between trichome and abnormal trichome soybeans (Figure 5), qRT-PCR was used to analyze the expression patterns of the nine *GmGeBP* genes. As can be seen from Figure 5, 66.7% of the *GmGeBP* (*GmGeBP1, GmGeBP2, GmGeBP4, GmGeBP5, GmGeBP8* and *GmGeBP9*) gene expression levels were upregulated in abnormal trichome soybean compared with trichome soybean. However, the expression levels of three *GmGeBP* genes (*GmGeBP3, 6* and *7*) determined via qRT-PCR analysis were weakly downregulated. We found that only *GmGeBP*4 was highly expressed in abnormal trichome soybean, while the other genes exhibited no obvious changes.

This section may be divided by subheadings. It should provide a concise and precise description of the experimental results, their interpretation and the experimental conclusions that can be drawn.

## 3. Discussion

According to the following observations, *GeBP* as a TF is predicted to play a role in the hormone pathway. First, the GeBP protein binds to the cis-regulatory element of the GLABROUS1 gene, which is an myb gene involved in epidermis cell determination and regulated by GA, a cytokinin hormone [6,14,15]. Secondly, the transcript levels of *GeBP* are positively regulated by BREVIPEDICELLUS (BP), a gene of the KNOTTED1 homeodomain (KNOX) family that positively regulates the cytokinin pathway in the shoot apical meristem (SAM) [16,17,18]. A preliminary analysis of the *GeBP* gene family has been performed in Arabidopsis and rice. However, this family has not previously been studied in soybean. Therefore, a systematic analysis of the *GmGeBP* gene family was performed and gene expression patterns were determined between abnormal trichome and trichome soybeans.

In the present study, nine of the *GmGeBP* gene family members were identified in the soybean genome using the bioinformatics method. It is known that segmental and tandem duplications played a role in the evolution and expansion of gene families in plants [19]. Gene duplication events were also identified for *GmGeBP* genes (Supplementary File S8). It was revealed that no *GmGeBP* member was identified as a tandem-duplicated event. In addition, four pairs of *GmGeBP* genes were found to be segmental duplicates. Here, 8 (88.9%) *GmGeBP* gene members were duplicated genes, which suggested that the gene duplication occupied a main position in the expansion of the *GmGeBP* family.

The analysis of the gene structure showed that the intron number of *GmGeBP* genes was one in *GmGeBP2* and *GmGeBP4*. It was reported that two *GeBP* genes in rice included one intron and two *GeBP* genes in tomato included seven introns and one intron, respectively. Consequently, our result indicated that the structures of GeBP genes were stable. Furthermore, three pairs of *GmGeBP* genes (*GmGeBP1* and *7*, *GmGeBP3* and *9, GmGeBP5* and *6*) exhibited similar intron–exon structures and intron numbers, and exhibited high conservation in the evolutionary process and high coherence with the characteristics defined in the above phylogenetic analysis (Figure 4). The phylogenetic analysis revealed that the *GeBP* genes from different plant species were separated by a high bootstrap value (95%). The analysis in Arabidopsis thaliana, rice and lucerne indicated that the genes were mainly classified into four groups and were consistent with the former studies. The majority of the GmGeBP proteins in the same group shared consensus motifs and similar exon–intron structures within the related genes.

Promoters in the upstream region of genes may provide useful information to further investigate the functions of *GmGeBP* genes in different developmental stages of plants due to their important roles involving the developmental or environmental regulation of gene expression [20]. Thus, the cis-elements in the promoter sequences were predicted using Soybase. The cis-elements of gene promoters in *GmGeBP* members were identified as having different functions, such as they could participate in the response to drought, heat, low temperature, anaerobic induction and defense stresses. Meanwhile, they were involve in the induction of GA, MeJA, auxin and ethylene. Among these cis-elements, *ABRE*, *TGACG-motif*, *TATC-box*, *TCA-element*, *ARE* and *AuxRE* were related to abscisic acid (ABA), MeJA, gibberlin, salicylic acid, anaerobic induction and auxin, respectively [21,22,23,24]. Therefore, these results will contribute to further understanding the various functional roles of *GmGeBP* genes in the formation of the trichome and in response to adverse conditions.

In order to reveal the potential functions of *GmGeBP* genes, the expression profiles of eight *GmGeBP* genes in different tissues were analyzed. According to the RNA-seq analysis, there existed three kinds of expression patterns among the nine *GmGeBP* genes, containing no expression, constitutive expression and tissue-specific expression patterns. During the different developmental stages of soybean, *GmGeBP4* and *GmGeBP8* showed little expression through all fourteen stages. Meanwhile, four genes (*GmGeBP1*, *5*, *6* and *7*) were continuously highly expressed in all tested tissues. Two genes (*GmGeBP3* and *9*) showed tissue-specific expression patterns, mainly expressed in the roots and nodules.

Previous studies have shown that *GeBP* genes are involved in the regulation of cell expansion in a CPR5-dependent manner, but not in the regulation of cell proliferation [25]. A specific phenotype of the *cpr5* mutant is the abnormal trichome development not found in the phenotype of any other constitutive pathogen response mutant [26,27]. In order to further clarify the role of the *GeBP* gene in the development of soybean trichome cells, we detected the expression of 9 *GmGeBP* genes between abnormal and normal trichomes via qRT-PCR. It has been reported that the *GEBP/GPL* gene plays an inhibitory role in cell expansion by offsetting the positive role of CPR5 in cell expansion [11]. In our results, there was one *GmGeBP* family member (*GmGeBP4*) displaying increased expression patterns in abnormal trichome soybean compared with trichome soybean. Thus, we could speculate that *GmGeBP4* might be related to the regulation of the CPR5-dependent processes, which are involved in the formation of trichome.

Taken together, we performed a genome-wide analysis of *GmGeBP* genes in soybean and obtained a series of information, including sequence information, gene duplication, conserved motif, gene structure and phylogenetic relationship data. The promoter analysis and expression patterns of *GmGeBP* genes in different tissues helped us better reveal the potential function of members in the *GmGeBP* gene family. Finally, the expression levels of *GmGeBP* genes in abnormal trichome soybean and trichome soybean provided clues for further studies of the roles of *GeBP* genes in the CPR5-related cell expansion process.

## 4. Materials and Methods

### 4.1. Plant Materials and Stress Treatment

The trichome soybean seeds “A3127” and abnormal trichome soybean seeds “Guoyu98-2” were provided by Yumin Wang. The seeds were germinated and grown in a controlled chamber (25 °C/20 °C, day/night, 16 h/12 h light/dark cycle) [28]. The leaves were collected and frozen in liquid nitrogen immediately and stored at −80 °C. Three biological duplications and three technical duplications of each experiment were performed.

### 4.2. Identification of GeBP Genes in Soybean

In this study, the Soybase database (http://www.soybase.org, accessed on 15 September 2021) was used to download the information for soybean *GeBP* genes, including the gene locations, gene sequences, CDS sequence data and protein information (Supplementary Files S1–S3) [13]. The physicochemical parameters were obtained from ExPASy (http://www.expasy.org/tools/, accessed on 15 September 2021). The ORF lengths, chromosome locations and numbers of exons and gene strands for each gene were obtained from the Phytozome database (http://www.phytozome.net/soybean.php, accessed on 15 September 2021).

Furthermore, potential *GeBP* genes from three other species were selected to analyze the evolutionary relationships among the different plant species, and these *GeBP* genes were confirmed using the same method above.

### 4.3. Subcellular Localization, Conserved Motifs and Gene Structure Analysis of GmGeBP Proteins

Firstly, the subcellular localizations of *GmGeBP* genes were identified using ProtComp9.0. Then, the conserved motifs of *GmGeBP* gene members were obtained from MEME version 4.11.0 (http://memesuite.org/tools/meme, accessed on 15 September 2021). The gene structure analysis was performed using a Gene Structure Display Sever (GSDS; http://gsds.cbi.pku.deu.cn/, accessed on 15 September 2021). The genes were identified as showing segmental duplication if they were found to exist on duplicated chromosomal blocks [29]. The paralogs were certified to be tandem-duplicated genes if the two genes were separated by five or fewer genes in a 100 kb region [30].

### 4.4. Chromosomal Location and Phylogenetic Tree Construction of GeBPs

Information about the chromosomal locations of *GmGeBP* genes was obtained from the Soybase database. The multiple sequence alignment of a total of 52 *GeBP* genes was performed with Clustal X2.0 software using default parameters [31], which were as follows: the gap opening and extension penalty of the pairwise alignment were 10 and 0.1, respectively; the gap opening and extension penalty of the multiple alignment were 10 and 0.2, respectively; the separation distance was 4. The phylogenetic tree was constructed with MEGA version 11.0 using the neighbor-joining (NJ) method and a bootstrap analysis with 1000 replications [32].

### 4.5. Promoter Analysis

Regions 2000 bp upstream of the translation start site of each *GmGeBP* gene were downloaded from Soybase (Supplementary File S4). Cis-elements in promoters of each *GmGeBP* gene were obtained using the PlantCARE server (Supplementary Files S5 and S6).

### 4.6. RNA-Seq Atlas Analysis

The transcription data for soybean *GeBP* genes were downloaded from the Soybase database. Here, fourteen soybean tissues were selected (root, nodule, seed 10-DAF, seed 14-DAF, seed 21-DAF, seed 25-DAF, seed 28-DAF, seed 35-DAF, seed 42-DAF, young leaf, flower, pod-shell10-DAF, pod-shell14-DAF, 1 cm pod). The heat map was produced using MeV v4.8 software (http://www.tm4.org/, accessed on 23 October 2021).

### 4.7. RNA Extraction and qRT-PCR Analysis

The total RNA was extracted from fresh samples using the Trizol method according to the manufacturer’s instructions. The quality of the RNA was certified via agarose gel electrophoresis before reverse transcription. The first-strand cDNA synthesis was performed as the instructions described. The gene-specific primers for *GmGeBP* genes were designed using Primer 5.0 (Supplementary File S7), and the raw data from the qRT-PCR analysis are shown in Supplementary File S9. The *GmActin* (LOC100792119) was used as an internal control. Three biological replicates were used per sample. Each sample was performed with three technical replicates. The relative expression level was calculated following the 2^−∆∆CT^ method [33].

## 5. Conclusions

In this study, we identified a *GeBP* gene family containing 9 *GmGeBP* genes in soybean, which were distributed on 7 of the 20 chromosomes. The GmGeBP proteins had three conserved motif patterns, indicating the conservation of its function. We also identified nine kinds of development-related cis elements and six kinds of hormone-responsive cis-elements in the putative *GmGeBP* gene promoters, indicating that the gene family is involved in multiple life processes. The trichome is a special epidermal cell, which protects plants from environmental stress and is an ideal model system for studying cell differentiation, cell cycle regulation and morphogenesis. In our results, one of the *GeBP* gene family members, GeBP4, was highly expressed in abnormal trichome soybean compared with normal trichome soybean, and was predicted to be involved in trichome development. Our study provides a theoretical basis for the functional study of *GeBP4* in trichome development in the future.

## Figures and Tables

**Table 1:** The information on the *GeBP* gene family in soybean.

				Location		Transcript Length	Protein					Subcellular Location	
	Name	Gene Identifier	Chromosome	Start	Stop	ORF Length (bp)	Lengh (aa)	pI	MW (kDa)	Extron	Gene Strand	WoLFPSORT Prediction	ProtComp9.0
1	GmGeBP01	Glyma03g245200	Gm03	44,249,778	44,251,845	2,068	405	5.00	44,614	1	reverse	nucl: 13, cyto: 1	NONE
2	GmGeBP02	Glyma05g088300	Gm05	18,609,493	18,613,137	3,483	387	9.08	43,231	2	forward	nucl: 9, chlo: 4, cyto: 1	NONE
3	GmGeBP03	Glyma10g160000	Gm10	39,422,646	39,424,482	1,837	372	4.75	41,286	1	reverse	nucl: 10.5, cyto_nucl: 6, cysk: 2, chlo: 1	NONE
4	GmGeBP04	Glyma10g160100	Gm10	39,434,399	39,435,558	1,128	375	5.20	43,151	2	forward	nucl: 5, cyto: 3, chlo: 2, mito: 2, plas: 1.5, golg_plas: 1.5	NONE
5	GmGeBP05	Glyma13g251000	Gm13	35,823,247	35,825,053	1,807	448	5.26	49,235	1	reverse	nucl: 11, plas: 2, cyto: 1	NONE
6	GmGeBP06	Glyma15g063300	Gm15	4,844,062	4,846,067	2,006	437	5.48	48,351	1	forward	nucl: 13, plas: 1	NONE
7	GmGeBP07	Glyma19g242600	Gm19	49,030,903	49,032,893	1,991	404	4.81	44,736	1	reverse	nucl: 14	NONE
8	GmGeBP08	Glyma20g228300	Gm20	46,272,228	46,273,452	1,225	357	4.93	41,057	1	reverse	cyto: 9, chlo: 1, nucl: 1, plas: 1, extr: 1, cysk: 1	NONE
9	GmGeBP09	Glyma20g228500	Gm20	46,281,417	46,282,947	1,531	353	4.65	39,647	1	forward	nucl: 9.5, cyto_nucl: 6, cysk: 2, cyto: 1.5, chlo: 1	NONE

bp, base pair; aa, amino acids; Da, Dalton. WoLF PSORT predictions: chlo (chloroplast), cyto (cytosol), nucl (nucleus), E.R. (endoplasmic reticulum), mito (mitochondria), plas (plasma membrane), extr (extracellular), cysk (cytoskeleton), plas (plasma membrane), vacu (vacuolar membrane). TargetP predictions: C (chloroplast), M (mitochondrion), S (secretory pathway), - (any other location); values indicate score (0.00–1.00) and reliability class (1–5; best class is 1).

## Data Availability

The data presented in this study are available on request from the corresponding author.

## Supplementary Materials

The following supporting information can be downloaded at: https://www.mdpi.com/article/10.3390/plants11141848/s1, Supplementary File S1: the genomic sequence information of soybean GeBP genes; Supplementary File S2: the CDS sequence information of soybean GeBP genes; Supplementary File S3: the proteins sequence information of soybean GeBP genes; Supplementary File S4: the sequence information of regions 2000bp upstream from the translation start site of each *GmGeBP*; Supplementary Files S5 and S6: Cis-elements in promoters of each *GmGeBP* gene; Supplementary File S7: The gene-specific primers for *GmGeBP* genes; Supplementary File S8: Pairwise identities between paralogous pairs of GeBP genes from Soybean; Supplementary File S9: Raw data of the first qRT-PCR results.

## References

[1] Kim E., Hwang S., Lee I. (2017). SoyNet: A database of co-functional networks for soybean *Glycine max*. Nucleic. Acids Res..

[2] Bohnert H.J., Gong Q., Li P., Ma S. (2006). Unraveling abiotic stress tolerance mechanisms-getting genomics going. Curr. Opin. Plant Biol..

[3] Curaba J., Herzog M., Vachon G. (2003). GeBP the first member of a new gene family in *Arabidopsis* encodes a nuclear protein with DNA-binding activity and is regulated by KNAT1. Plant J..

[4] Berhin A., Nawrath C., Hachez C. (2022). Subtle interplay between trichome development and cuticle formation in plants. New Phytol..

[5] Ma C., Chen Q., Wang S., Lers A. (2021). Downregulation of GeBP-like alpha factor by MiR827 suggests their involvement in senescence and phosphate homeostasis. BMC Biol..

[6] Perazza D., Vachon G., Herzog M. (1998). Gibberellins promote trichome formation by Up-regulating GLABROUS1 in arabidopsis. Plant Physiol..

[7] Jakoby M., Weisshaar B., Droge-Laser W., Vicente-Carbajosa J., Tiedemann J., Kroj T., Parcy F. (2002). bZIP transcription factors in Arabidopsis. Trends Plant Sci..

[8] Huang J., Zhang Q., He Y., Liu W., Xu Y., Liu K., Xian F., Li J., Hu J. (2021). Genome-Wide Identification, Expansion Mechanism and Expression Profiling Analysis of GLABROUS1 Enhancer-Binding Protein (GeBP) Gene Family in Gramineae Crops. Int. J. Mol. Sci..

[9] Chevalier F., Perazza D., Laporte F., Le Henanff G., Hornitschek P., Bonneville J.M., Herzog M., Vachon G. (2008). GeBP and GeBP-like proteins are noncanonical leucine-zipper transcription factors that regulate cytokinin response in Arabidopsis. Plant Physiol..

[10] Khare D., Mitsuda N., Lee S., Song W.Y., Hwang D., Ohme-Takagi M., Martinoia E., Lee Y., Hwang J.U. (2017). Root avoidance of toxic metals requires the GeBP-LIKE 4 transcription factor in *Arabidopsis thaliana*. New Phytol..

[11] Perazza D., Laporte F., Balague C., Chevalier F., Remo S., Bourge M., Larkin J., Herzog M., Vachon G. (2011). GeBP/GPL transcription factors regulate a subset of CPR5-dependent processes. Plant Physiol..

[12] Xu G., Guo C., Shan H., Kong H. (2012). Divergence of duplicate genes in exon-intron structure. Proc. Natl. Acad. Sci. USA.

[13] Grant D., Nelson R.T., Cannon S.B., Shoemaker R.C. (2010). SoyBase, the USDA-ARS soybean genetics and genomics database. Nucleic. Acids Res..

[14] Oppenheimer D., Herman P., Sivakumaran S., Esch J., Marks M. (1991). A myb gene required for leaf trichome differentiation in Arabidopsis is expressed in stipules. Cell.

[15] Gan Y., Liu C., Yu H., Broun P. (2007). Integration of cytokinin and gibberellin signalling by Arabidopsis transcription factors GIS, ZFP8 and GIS2 in the regulation of epidermal cell fate. Development.

[16] Curaba J., Moritz T., Blervaque R., Parcy F., Raz V., Herzog M., Vachon G. (2004). AtGA3ox2, a key gene responsible for bioactive gibberellin biosynthesis, is regulated during embryogenesis by *LEAFY COTYLEDON*_2_ and *FUSCA*_3_ in Arabidopsis. Plant Physiol..

[17] Jasinski S., Piazza P., Craft J., Hay A., Woolley L., Rieu I., Phillips A., Hedden P., Tsiantis M. (2005). KNOX action in *Arabidopsis* is mediated by coordinate regulation of cytokinin and gibberellin activities. Curr. Biol..

[18] Yanai O., Shani E., Dolezal K., Tarkowski P., Sablowski R., Sandberg G., Samach A., Ori N. (2005). *Arabidopsis* KNOXI proteins activate cytokinin biosynthesis. Curr. Biol..

[19] Vision T., Brown D., Tanksley S. (2000). The origins of genomic duplications in Arabidopsis. Science.

[20] Zhu Y., Wu N., Song W., Yin G., Qin Y., Yan Y., Hu Y. (2014). Soybean (*Glycine max*) expansin gene superfamily origins: Segmental and tandem duplication events followed by divergent selection among subfamilies. BMC Plant Biol..

[21] Sakuma Y., Liu Q., Dubouzet J.G., Abe H., Shinozaki K., Yamaguchi-Shinozaki K. (2002). DNA-binding specificity of the ERF/AP2 domain of Arabidopsis DREBs, transcription factors involved in dehydration- and cold-inducible gene expression. Biochem. Biophys Res. Commun..

[22] Maruyama-Nakashita A., Nakamura Y., Watanabe-Takahashi A., Inoue E., Yamaya T., Takahashi H. (2005). Identification of a novel cis-acting element conferring sulfur deficiency response in Arabidopsis roots. Plant J..

[23] Shi Z., Maximova S.N., Liu Y., Verica J., Guiltinan M.J. (2010). Functional analysis of the *Theobroma cacao* NPR1 gene in *Arabidopsis*. BMC Plant Biol..

[24] Osakabe Y., Yamaguchi-Shinozaki K., Shinozaki K., Tran L.P. (2014). ABA control of plant macroelement membrane transport systems in response to water deficit and high salinity. New Phytol..

[25] Xia Y., Yu K., Navarre D., Seebold K., Kachroo A., Kachroo P. (2010). The glabra1 mutation affects cuticle formation and plant responses to microbes. Plant Physiol..

[26] Kirik V., Schnittger A., Radchuk V., Adler K., Hulskamp M., Baumlein H. (2001). Ectopic expression of the Arabidopsis AtMYB23 gene induces differentiation of trichome cells. Dev. Biol..

[27] Brininstool G., Kasili R., Simmons L.A., Kirik V., Hulskamp M., Larkin J.C. (2008). Constitutive Expressor of Pathogenesis-related Genes5 affects cell wall biogenesis and trichome development. BMC Plant Biol..

[28] Zhang L., Zhao H.K., Dong Q.L., Zhang Y.Y., Wang Y.M., Li H.Y., Xing G.J., Li Q.Y., Dong Y.S. (2015). Genome-wide analysis and expression profiling under heat and drought treatments of HSP70 gene family in soybean (*Glycine max* L.). Front. Plant Sci..

[29] Schauser L., Wieloch W., Stougaard J. (2005). Evolution of NIN-like proteins in *Arabidopsis*, rice, and *Lotus japonicus*. J. Mol. Evol..

[30] Tang H., Wang X., Bowers J.E., Ming R., Alam M., Paterson A.H. (2008). Unraveling ancient hexaploidy through multiply-aligned angiosperm gene maps. Genome Res..

[31] Thompson J., Gibson T., Plewniak F., Jeanmougin F., Higgins D. (1997). The CLUSTAL_X windows interface: Flexible strategies for multiple sequence alignment aided by quality analysis tools. Nucleic. Acids Res..

[32] Tamura K., Dudley J., Nei M., Kumar S. (2007). MEGA4: Molecular Evolutionary Genetics Analysis (MEGA) software version 4.0. Mol. Biol. Evol..

[33] Livak K.J., Schmittgen T.D. (2001). Analysis of relative gene expression data using real-time quantitative PCR and the 2(-Delta Delta C(T)) Method. Methods.

