# Supplementary material for: Genome-Wide Characterization and Expression Analysis of GeBP Family Genes in Soybean
Source: Plants (Basel). 2022 Jul 14;11(14):1848. doi: 10.3390/plants11141848 (PMC9318833; doi:10.3390/plants11141848)
Supplement: Supplementary file 1 [file plants-11-01848-s001.zip › Supplementary file 1.pdf]

>GmGeBP1
ATGGCTTCGGAGCAACACGACGCCGTTTTCCGCGAAGAAGACATGGACGACGACGACGAC
GAGTCTCAGGAGGACGAAGAGTACGAAGAAGACGACGACGAAGAAAACGTGCCCTCTCCC
TCCACCGCTCTCGCTGTCACCGTCGCCGTCCCCGGTTCCGCCGTCTCCAACGGCGGCGGT
GGCTCTCCAATTTCGAAGCCCACCGCCACCGCCACCACCGCCACCATCGTCCTCGCCGAC
TCATCCGATCCGAAGCGGCGTCGCCTGGAGCTAATCGAGGAGAAAAAGCCGCCGCCGCCG
CTGGATGACTCGCGGCGGCTGTTTCAGCGGCTGTGGACCGATGAGGATGAGATCGAGCTC
TTGCAAGGGTTCCTCGACTACACATCGCAGCGAGGATCCTCGCACCACAACGACACCGCT
TTGTTCTACGACCAAATCAAGTCGAAGCTCCAACTCGATTTCAACAAGAATCAGCTCGTG
GAGAAGATCCGAAGGCTGAAGAAGAAGTACCGGAACGTCCTCAACAAGATTTGCTCCGGC
AAGGAATTCTCCTTCAAGAGCGCTCACGATCAAGCCACCTTCGAAATCTCGCGCAAGATC
TGGAGCAACGTGACTCCAGTCGGCGACAATTCATTGGACGACGACGAAATCAACCCTAGC
CGTAGCCCCAACCCTAACCTTAATTTTAGCCCTGTAATTCTTAAGAATGAAACGATTTTC
AGGAATTCCACGGAGAAGAAAACACCGAAACGCTCTCGGCCTCGATCGGCGGTGAAAATC
GAGCCAAATGACGGATCCGCGTCGAACAGAGATCATAATTGTATTAGCAATACTACACCT
ACTGCTACTGCTGCTGCTACTAACACTACTGCTGCTGCTGCTGCTGGTACTAACAATAAT
AACTGTAACAGTGGTTATGGAAATAACATACCGAGTTTGATTGAGGAGACGGTGAAGAGT
TGTTTGTCGCCGGTGTTGAAGGAATTGATGGCAGGGGCCATGGGAGGAGGAGCGTTTGGA
GGGAGAGGGTTTTCGTTGAACTTGAACCCTATGCCTTTTATGAATTTGAGTTTTGGCGGT
GGGGAAATGGTGGATGAGAAATGGAGGAAGCAACAGATATTGGAGTTGGAAGTGTATTCG
AAGCGGTTGGAACTGGTGCAGGATCAGATCAAGGCAGCTATGGAGGAGTTGCGGTCACAT
GGAGGAGGAGGATTATAG
>GmGeBP2
ATGATGTCAAGTGACCGGATCACCCTTAAAATTGGTCCGCCTTTACTATTTTCTTTTCCC
GGCGAAGTCCGCGGTTTCTATTTGGTATCTGCATTTCTCCACCAGAAGCAGCTACCTCCC
CCCATTGAGGCCTCTTCCGATGAAGAGCAACGACCTTCCTCCAAGCAACACACAGAAGAA
GGAGTTTCCTCTTCAGAAGAAGCTTCTTCCCAAGAAGAAGACGACGACGACCAACCTCCC
ACCCTTCCTCTCGCTTCCGCAAACCCCCACCCGAAACCGTCGTCCTCCGACTCCGACACC
GACTTCGAGCCCACCAAAGTAAAACCCAAGCCCACGGACCAGGCCCAGAAGCCCCAGCCC
TCACCCGCGCCGCCCAAATGGGGATCCAAGCGCCCTGCCCAGAACAACGCCCCTGCCACT
GACCCGAAACGCGCGAAGAAGAAATTGACCAATTCTTCTTCCGCCGCTGCCGCCCACGAG
ACGGAGGAGAAGTCCGGTGGCGGCCAAGCGAAGTTGTCTCAGAGACTCTTCAGCAAGGAA
GACGAACTCGCCATTCTTAAGGGCATGGCTGAGTTCATTTCGAAGACGGGCCAAGACCCT
TACAAGTACGCCGATGCCTTCCAAAATTTCGTTAAGAATTCGCTCCGCGTGGAGGCTTCG
AGCAACCAGATCAAGGAGAAGATCCGACGGCTGAAGAAGAAGTTTGAAACTAAAGCACAG
AGAGCGAAGAAATGGGAGGACCCTGAGTTCTCCAAATTTCACGACCGGACTGTGTTTGAA
TTATCAAAGAAGGTTTGGGGAGAAGGAGCCAATGGGCTGGTGGAGAAGCCAAAACCTAAT
AATGGAAAAAGAAAAACTGCCAAGACTCCAAAGAAGGATGCTACTAGCAGGAATGTAGTA
GCAAAATCTGAAACTACAACACTACTGGAGTCGATGGAGTTGGAGGAGTGTGGGAATGTA
AATTTGCTTTACCGCGAAGTATCTGGTTTCAAAGAACTGAATGAGGATGAGATGAAGAGG
GGATTGGCGTTAATTGGAGAATCTAAGAGGAAAGAGTTGGAGGGCAAGTGGAGGAAATTG
CGACTTGCTGAGATGGAACTGGTTGCGAATCGCTCACTACTTATTGGGGAGCAGATTAAG
TTGATATTTGAAGCACTTCAGTAA
>GmGeBP3

ATGGAATCCGATCTAAACGACGCCGTTTTCCCCGAAGAAGATCTCGACGACGACGACGAC
GATGAAACTCCAGAGGACGAAGAGGACTACGACGACGAAACCGAACCCCCTCCCTTCGTC
CTCGCCGTCGTAGCAGTCGCGCCGCCCGCTTCCACCGCCTCCGAAACACTCGACACCACG
TTGATCCCGATCTCCGCCGTCGCCGATTCCTCACTGAAACCACTGCACCCGGAGCTAATC
GAGGAGAAAAAAGCGCTGGACGATTCGCGGCGACTGTTCCAGCGTCTGTGGACGGACGAG
GACGAGATCGGGCTCTTGCAGGGGTTTCTGGACTACACGGCGCAGCGAGGATCCTCTCAC
CACAGCGATACCGCCTTGTTCTACGACCAAATCAAGTCGAAGCTCCAACTCGGCTTCAAC
AAGAACCAGCTCGTCGAGAAGCTCCGAAGGCTGAAGAAGAAGTACCGCAACGTCGTCACC
AAAATCAGCTCCGGCAAGGATGTCTCCTTCAAGAGCCCTCACGACAAAGCCACCTTCGAA
ATCTCACGCAGGATATGGAGCAACACCGCTCCAATAAGCGGTCCCGTCGAAGACGACGAC
GAAATCAACCCTAACCCTAATTTCGGCAATTCCGCGAAGACACCTATCTCGCGGAAGCGG
TCGCGGCCTCAGAAGCGCGAGTTGAACGACGGTTCAACATTGAATAGAGATAATAATTGT
ATTGGCAATAATAATAATAATAGCAATAGTAATAATAATAATAATGAGAATTGTAATGGT
AGGCATAACTTACAGGGTTTGATTGAAGAGACGGTTAAGAGTTGCGTGTCGCCGGTGTTG
AAGGAGTTGGTGTGTGGTACCGGTGGCATGGAATTGGGAAGAGGGTTTGGAGTTGGAGGA
GGGCTTGGAGTTGGAGGAGGGCTTGCATTGAATTCCTTGCAAACTCAAATGACAATGCCA
ATGACTTTATTGAATTTGAGGATTGGGGAAACAACGATGGATGAGAAGTGGAGGAAGCAA
CAGATTTTGGAGCTGGAAGTGTATTCCAAGCGTTTGGAATTGGTGCAGGATGAGATCAAG
GTTGCTCTCGAGGAGTTGCGATCAGCTGGAGGAGTATAA
>GmGeBP4
ATGCACAACTGCCATAAACGAACAAACGGCTACCTTGACACTCTAATATGCGAAAAAAGG
AAATGTGATATCTCCTCAACAATGCTTTCCCGGTTGGTTTCTTGGATCTTGTCGCCCTCC
TCTTCTGAAGAAGAAGAAGAAATCCTTGACATCATCAAAGATAATGACATTAACCATGAA
AACGACCAAAAGTTCAACGTAGAAGATGACAAGAACGACCATTTTCTTAACTCATGCGAT
GTGGACGACACCATTCCCATTGCACTTGCCGTTCCCAATGCCTCCCCCGCCGTGACAGTG
GCCTTCCCGGCCAATGACGAAAGAAACACCGTCCCAACCACCGCCACTGTGGCCACTATA
GTAACACGCTCAAAAGGGCAGCGCAACGCCAAGTATTCCGGAATGGTGAGACAATACCAA
AGGCTGCGGACGAAGGAGGATGAGATGGAACTGTTGAAGGGATACCTTGATTACGTCAAG
CAGCATAGAAAGGAAACCACCACCCTTCTTTACGTCGTAGTTTCATGTATGATCACAAAC
CAGCTTGTTGAGAAACTGCGTAGGTTAAAGAGAAAACACAAGTTGGCTTTGGAAAAAGGC
AAGGACAAGGAGGTTCCCTTTAGGAACCCCCAGGAGCAGGCCATTTTTGAAATTTCCCAC
AAGATTTGGGCCAATGACACAGATAATATAATAGTCCAAGATGCCTTGGATGGTGATGAA
TCAGGACACACTCCTGAAAGTCATGATCATGTTGGCAACGTCAAGGTGAAGATTGAACAA
GTTGACAACAGTGATGAAATAGGCAATAGAGTGCCAAAGCGGTTGCGGCTAGATGATGCA
GACGATATGAACAGAACAAATGACCAGAACAATGGTGATAGCATACAAGGCTTCATTGAG
GACACCATGAGGTCCTGTTTCTCACCATTGCTGAAGGAAGTGTTGGATGAAGCACAGGAA
GAGTCACTTCCTGAGTTGGAGGCAATCCCAATGCCGCTATGCTCCGGGGAAGTGGACCAT
GAACAATGGAGAAAACGGAGGATTTTGGAGTTGGAGGTGTATGTGAAGCGGTTGGAGTTG
TTGCAGAATCAGATCAAGTCTAGATTGGAAGAGTTGCGATCTAGCTGA
>GmGeBP5
ATGGCACAGAAGCAAAAGCTGCGCCCTTCTCCTCTCGACGAGCCACCCACTGCTTCCTCT
TCCGATTCCGAGGAAGAAGAGCCACAACAACAACAACCATCCTCCCAGAAGAACAAAGAA
GAGGAAGACGAAGAAGTTTCCTCCGGAGAAGAAGAAGAAGAAGACGAAGAAGAAGAAGAA
GCTGCTTCCTCCGAAGAAGAGGAAGAAGACGAAGATCTCCCACCACCACCAGTTTCCAAA

AACCCTCCACCCCCTCCCGCAAACCCTCAGCCCCAACACTCTTCCTCCGAATCCGAAACC
GAATCAGGATCCGAGACCGAATCCGAACCTGACCCCACTCCCGTCAAGGTCAAGCCTTTA
GCTTCCAAGCCCATGGACCAGGCCCAAAAGCCCAAGGCCCAGCCCTCCCCGGCGCCACCC
CCAAAATTGACACTCAAGCGCCCCGCCGAGAACAACAACAACAACGCCCGCGTCGCCGAC
TCCAAACGCGCCAAGAAGAAAGCCACCGAATCTTCTTCTGCCGCCAACTCCGCCGCGGCC
GCTGCTTCTGACGACGAGATGGAGGAGGACGGGAAGAAGTCCGGCGATAACTCGAAGAAG
TTTCAGAGACTGTGGAGCGAGGAGGACGAGCTCGCCATTGTGAAGGGCGTGGTTGAGTTC
ACTTCGAAAACAGGGCTGGACCCTCTTAAGTTTCCCAATACCAACGCTTTTCACGATTTC
GTGAAGAAGTCGCTTCACGTGGAAGTTTCCTGCAACCAGCTGAAGGAGAAGGTCCGAAGG
CTCAAGAAGAAGTTTGAGACCCAGGCTGGAAAAGGGAAGAACGGAGAGGCCCCTAAGTTT
TCCAAACCGCACGATCAGAAATTCTTTGAATTGTCCAAAAAGGTTTGGGGACGTGAGGTT
ACTGCTGGAGCTAATGGGGGCCCCGTGGAGAAGCCCAAGTCTAATGGGAGTGCTGTCAAG
AGTCCGAAGAAGAAGGAAAGCGGTAGCAGGAATGTGGCTTCTGCTAAGAAACCGAAGCCT
GAATCAAAACCGGAGCCGGTTCCAGTGCTGTCCTTGGAGTATAAGGATTCTGAAAAGATG
CAGATTAATCAAAAGCCTGATGGTGGTGATGCAAGTTTGTTTTTGCGCGAATTGGCTCGA
TCCAAAGAGGGTGCGAGTATTTGTAAGCTGGATGAGGATGATGTGAAGAGGGGGTTGGAG
TTGATTGGAGAGTCAAAGAGGGCAGAGTTGAGGGGGAAGTGGAAGAAATTACATCTTGCT
GAGATGGAACTGTTTGCGAATCGCTCAGAACTGATAGGGGAGCAGACTAAGTTGATACTT
GAGGCGCTTCAGGCATCCGATCATTAG

>GmGeBP6

ATGGCTCAAAAACAAAAGCTGCGCCCTTCTCCTCTGGACGAGCCACCCACCGCTTCCTCT
TCCGATTCCGAGGAAGAAGAGGAGCAACAACAACAACAACCATCCTCTCAGCAACACGAA
GAAGAGGAAGAAGAAGTTTCCTCCGGAGAAGAAGAGGAAGCTTCCTCCGAAGAAGAGGAA
GACGAAAATCTCCCCCCACCACCCATCTCCAAAAACCCTCCACCCCCTCCTCCCTCAAAC
CCTCAGCCCCAACCCACTTCCTCCGAATCCGAAACCGAATCGGGTTCCGAAACCGAATCA
GAACCCCACCCCACTCCCGTTAAGGTCAAACCCCTAGCCTCCAAGCCCATGGACCAGGCC
CAAAAGCCCAAAGCTCAACCCTCCCCGGCGCCGCCGCCGCCAAAATCGGCATCCAAGCGC
CCCGCCGAGAACAACAACAACAACGCCCGTGTCGCCGACCCCAAACGCGCGAAGAAAAAA
GCCACCGAATCTTCTTCTGCCGCCGCCATCTCCGACGACGAGATGGAGGAGGACGGGAAG
AAGTCCGGCGATAACTCGAAGAAGTTTCAGAGACTGTGGAGCGAGGAGGACGAACTCGCC
ATTCTGAAGGGCGTGGTTGAGTTCACATCGAAAACAGGGCTGGACCCTCTTAAGTTCCCC
AATGCCAACGCTTTCCACGATTTCATGAAGAAGTCGCTTCACGTGGAATTTTCCAGCAAC
CAGCTGAAGGAGAAGCTCCGAAGGCTCAAGAAGAAGTTTGAGACCCAGGCAGGAAAAGGG
AAGAATGGAGATGCCCCTAAGTTTTCCAAACCGCACGATCAGAAATTCTTTGAATTGTCC
AAAAAGGCTTGGGGAAGTGAGGATGGTGGTGTAGCCAATGGCTCCGTGGAGAAGCCCAAG
TCCAATGGGAATGCTGCCAAGAGTCCGAATCCGAAGAAGAAGGAATCCGGTAGCAGGAAT
GTGGCTTCTGCTAAGAAACCGAAGCCTGAAACAAATCCGGAGCCGGCGCCGGTGCCGTCC
TTGGAGTTTAAGGAGTCTGAAAGGATGGAGATTGATCAAAAGCCTGATGGTGGTGATGCA
TGTTTGTTTTTGCGCGAATTGGTTCGATACAAAGAGGGTGCCAATGTTTCTAGGCTGGAT
GAGGATGATGTGAAGAGGGGGTTGGAGTTGATTGAGGAATCAAAGAGGGCAGAGTTGAGG
GGGAAGTGGAAGAAATTACATCATGCTGAGATGGAACTGTTTGCGAATCGCTCAGAACTG
ATTGGGGAGCAGACTAAGTTGATACTCGAGGCGCTTCGGTCATCCAATCATTAG

>GmGeBP7

ATGGCTTCTCAGCAACACGACGCCGTTTTCCGCGAAGAAGACATGGACGACGACGACGAC

GAGTCTCAGGAGGACGGAGACTACGAAGAAGAAGACGACGACGTTTTGGCCGACGACGAA
GAAAACGAGCCCTCTCCCTCCACGGCTCTTGCCGTCACCGTCGCCGTCCCCGGTTCCTCC
GTCTCCAACGGCGGTGCCGCTCCAATTTCGACGCCCACCGCCACCACCATCGTCGTCGCA
GACTCCTCCGATCCGAAGCGGCGGCGCCTCGAGCCAGTCGAGGAGAAAAAGCCGCCGCCG
ACGCCGGACGACTCGCGGCGGCTGTTTCAGCGGCTGTGGACCGACGAGGACGAGATCGAG
CTCTTGCAAGGCTTCCTAGACTACACCTCGCAGCGAGGATCCTCGCACCACAACGACACG
GCTTTGTTCTACGACCAAATCAAGTCGAAGCTCCAACTCGATTTCAACAAGAACCAGCTC
GTCGAGAAGATCCGAAGGCTGAAGAAGAAGTACCGAAACGTCCTCAACAAGATTGGCTCC
GGCAAGGAATTCTCCTTCAAGAGCGCTCACGATCAAGCCACCTTCGAAATCTCGCGCAAG
ATCTGGAGCAACGTGACTCCAATCGGCGACAATTCCTTGGACGACGACGAAATCAACCCT
AACCGTAGCCCCAACCCTAACCTTAATTTTAGCCCTATAATTCTTAAGAATGAAATGATT
TTCAGGAACCCCGCGGAGAAGAAAACACCGAAACGCTCTCGGCCACGATCGGCGGTGAAA
ATCGAGCCAAATGACGGATCCGCGTCGAACAGAGATCATGATTGTATTAGCAATGCTACG
CCTACTGCTACTGCTACTGCTGCTGCTACTAACACTACTCCTACTGCAGCTACTACTAAT
GATAACTGTAACAGTGGTTATGGAAATAACATACCGAGTATGATCGAGGAGACGGTGAGG
AGTTGTTTGTCGCCGGTGTTGAAGGAGTTGATGGCAGGGGCCATGGGAGGAGGAGCGTTT
GGAGCGAGAGGGTTTTCGTTGAACTTGAACCCTATGCCTTTGATGAATTGGAGTTTTGGC
GGTGGGGAAATGGTGGATGAGAAATGGAGGAAACAACAGATATTGGAGTTGGAAGTGTAC
TCAAAGCGGTTGGAGCTGGTTCAGGATCAGATCAAGGCTGCTATGGAGGAGTTGCGGTCA
CACGGAGGAGGTTGA
>GmGeBP8
ATGCTTTCCCCGTTGGTTTCTTGGATCTTGTCTCCCTCCTCTTCCTCCTCCTCTTCCGAA
GAAGAAGAAGAAGAGATCCTTGACATTATCACAGATAATGACATTAACCATGAAAATGAC
CAAAAGCTCAATGTAGAAGATGACGAGTGTGATGTGGACAACACCATTCCCGTTGCACTT
GCTGTTCCCAATGCCTCCCCCGCCGTGACAGTGGCCTTCCCGGCCAATGACGAAAGAAAC
ACCATCCCAGTCACCGCCACTGCCACCACTGTCGTAACATGCTCAAAAGGGCAGCGCAAC
GCCAAGTATTCCGGAATGGTGAGACAATACCAAAGGCTGTGGACGAAGGAGGATGAGATG
GAACTGTTGAAGGGATACCTTGATTACGTCAAGCGGCATAGAAAGGAAACCACCACCCTC
CAAAGCGTCGTAGCCTCGTTGTATGATCACGTAAGGCCCAAACTGAACGTGAGTTTCAAC
AAAAATCAGCTTGTTGAGAAACTGCGTAGGTTAAAGAGAAAACACAAGTTAGCTTTGGAC
AAAGACAAGGACAAGGAGGTTCCCTTTAGAAACCCCCAGGAGCAGGCCATTTTTGAAATT
TCCCACAAGATTTGGGGCATTGACACGGATAATATAATAGACCAAGATGCCTTGGATGGT
TATGAATCAGGACACACTCCTGAAAGTCATGATCATGTTGGCAACATCAAGGTGAAGATT
GAACAACTTGACAACAATGATGAAATAGATAATAGAGTGCCAAAGCGGTTGCGGCTAGAT
GATGCAGATGATGTGAACAAAACAAATGACCAAAACAATGGTGATAGCATACAAGGCTTC
ATTGAGGAGACCATGAGGTCATGTTTCCCACCATTGCTGAAGGAAGTGTTGCATGATGCA
CATGAAGAGCCACTTCCTGAGTTGGAGCCAATCCCAATGCCACTATGCCCTGGGGAAGTG
GACCATGAACAATGGAGAAAACGGAGGATTTTGGAGTTGGAGGTGTATGTGAAGCGGTTG
GAGTTGTTGCAGGATCAGATCAAGTCTAGATTGGAAGAGTTGCGATCTAACTGA
>GmGeBP9
ATGGAATCCGATCTAAACGACGCCGTTTTTCCCGAAGAAGATCTCGACGACGACGACGAA
ACTCCAGAGGATGAAGAGGAGGAGGAGGACGACGACGTCTTAGATGACGATGAAACCGAA
CCCCCTCCCTCCGTCATCGCCGTCGCGCCGCCCGCTTCCGAAACGCTCGACACCGCGTTA
ATTCCGATCTCCTCCGTCGCGGATTCCTCGCCGAAACCGCTGCGCACGGAGCTAATCGAG

GAGAAAAAAGCGCTGGACGATTCGCGGCGACTGTTCCAGCGTCTGTGGACAGACGAGGAC
GAGATCGGGCTCTTGCAGGGTTTTCTGGAGTACACGGCGCAGCGAGGATCCTCTCACCAC
AACGACACCGCCCTGTTCTACGACCAAATCAAGTCGAAGCTCCAACTCGGCTTCAACAAG
AACCAGCTCGTCGAGAAGCTCCGAAGGCTGAAGAAGAAGTACCGCAACGTCCTCAACAAA
ATCAGCTCCGGCAAGGAAGTCTCTTTCAAGAGCCCTCACGACCGAGCCACCTTCGAAATC
TCGCGCAGGATCTGGAGCAACACCGCTCCAATCACCGGTCCCGTCGAAGACGACGACGAA
ATCATCACTAACCCTAATTTCGGCAATTCGGCGAAGATGCCTATTTCGCGGAAGCGGTCG
CGGCCTCAGAAGCGCGAGTTGAACGACGGTTCAACGTTGAATAGAGATAATAATTGCAAT
AGTAACAGTAATAATAATAATAATAATGAGAATTGTAATAGTAGGCTTAACTTACAGGGT
TTGATTGAAGAGACGGTGAGGAGTTGCGTGTCGCCGGTGTTGAAGGAGTTGGCGTGTGGT
ACTGGTGGCATGGGATTGGGAAGAGGGTTCGCATTGAATCCCTTGCAAATGCCAATGCCA
ATGAGTTTAATGAATTTGGGGATTGTTGGGGAAACGGCGATGGATGAGAAGTGGAGGAAG
CAACAGATTCTGGAGCTGGAAGTGTATTCCAAACGTTTGGAATTGGTGCAGAATGAGATC
AAGGTTGCTCTCGAGGAATTGCGTTCAGCTGGTGGAGGATGA
