# Supplementary material for: Genome-Wide Characterization and Expression Analysis of GeBP Family Genes in Soybean
Source: Plants (Basel). 2022 Jul 14;11(14):1848. doi: 10.3390/plants11141848 (PMC9318833; doi:10.3390/plants11141848)
Supplement: Supplementary file 1 [file plants-11-01848-s001.zip › Supplementary file 2.pdf]

>GmGeBP1

AAAAATTTATAAAGAATCAGGAGATTAATTTAACTTAGAATATTTTTGTTCTCTAAATAA
CATTATTAAGTATTTGAAAATGTAGAGGATAGTTTTTAATACGATAAAAAAAATAAAAAA
TGTAAGTGCAGCGTTGCGTTTGTGGCGAAAGAAGAGGTACATTCCTTGTAAAATAAAAAT
AATACGGGGGGTTTGGTTGGTCCACTAGGCTACCTAAACTAAACTAAAAACCAAAACCCA
AAACCAGCCTTGGGATTCCCCCATTACCAGCAGCGAAGTAGAGGTGAGTTTGATTGAGTC
AGTCCAACAATAACTAAAAACCCGTGACTCAGCCCACTCTTGCTTTGCTTGGCCTAACCG
CTAACCAAACCAAAGCTCTCTCAATGGCTTCGGAGCAACACGACGCCGTTTTCCGCGAAG
AAGACATGGACGACGACGACGACGAGTCTCAGGAGGACGAAGAGTACGAAGAAGACGACG
ACGAAGAAAACGTGCCCTCTCCCTCCACCGCTCTCGCTGTCACCGTCGCCGTCCCCGGTT
CCGCCGTCTCCAACGGCGGCGGTGGCTCTCCAATTTCGAAGCCCACCGCCACCGCCACCA
CCGCCACCATCGTCCTCGCCGACTCATCCGATCCGAAGCGGCGTCGCCTGGAGCTAATCG
AGGAGAAAAAGCCGCCGCCGCCGCTGGATGACTCGCGGCGGCTGTTTCAGCGGCTGTGGA
CCGATGAGGATGAGATCGAGCTCTTGCAAGGGTTCCTCGACTACACATCGCAGCGAGGAT
CCTCGCACCACAACGACACCGCTTTGTTCTACGACCAAATCAAGTCGAAGCTCCAACTCG
ATTTCAACAAGAATCAGCTCGTGGAGAAGATCCGAAGGCTGAAGAAGAAGTACCGGAACG
TCCTCAACAAGATTTGCTCCGGCAAGGAATTCTCCTTCAAGAGCGCTCACGATCAAGCCA
CCTTCGAAATCTCGCGCAAGATCTGGAGCAACGTGACTCCAGTCGGCGACAATTCATTGG
ACGACGACGAAATCAACCCTAGCCGTAGCCCCAACCCTAACCTTAATTTTAGCCCTGTAA
TTCTTAAGAATGAAACGATTTTCAGGAATTCCACGGAGAAGAAAACACCGAAACGCTCTC
GGCCTCGATCGGCGGTGAAAATCGAGCCAAATGACGGATCCGCGTCGAACAGAGATCATA
ATTGTATTAGCAATACTACACCTACTGCTACTGCTGCTGCTACTAACACTACTGCTGCTG
CTGCTGCTGGTACTAACAATAATAACTGTAACAGTGGTTATGGAAATAACATACCGAGTT
TGATTGAGGAGACGGTGAAGAGTTGTTTGTCGCCGGTGTTGAAGGAATTGATGGCAGGGG
CCATGGGAGGAGGAGCGTTTGGAGGGAGAGGGTTTTCGTTGAACTTGAACCCTATGCCTT
TTATGAATTTGAGTTTTGGCGGTGGGGAAATGGTGGATGAGAAATGGAGGAAGCAACAGA
TATTGGAGTTGGAAGTGTATTCGAAGCGGTTGGAACTGGTGCAGGATCAGATCAAGGCAG
CTATGGAGGAGTTGCGGTCACATGGAGGAGGAGGATTATAGTTTGTTTTTGGTTTTGTAG
CGAGTGATTGGAGTTCTTCTTGCTCTTCGTGTTCACTTCATCATACGGATTTGCTTGGTA
ATTTATCCATCAATGGTTGGTTCATACTCATAGCTTAGAAGTTAGCTAGTCGTAGTAACT
TGTAAGTGGTAACTAGTGTTTTTCTTTTCGGTTTTAGCTTTCGCTGATTCGGTAGAGCAC
TTGTCCATTAAAATAAAAATGTTTTATTTTATATTTTAGCCGTTTTTATGTTCCTTTAAA
TTTCAATGGTCTCATACATGTACAGAACAGATTGAAATAATTGGTGCGGAGTGATGGCTT
TGTACCATTATATATATGTTGTAAAAGATGCGAAAGTAGTGGGTTCTCAATTGTGCAACG
TTCTTCCTGAGCACTTTGTTCTTAAAAGTATATCTTTCGTATTTTTATGATTCAATTGAT
TGTTAAAGATGGCATTTTCTTATCTGAG

>GmGeBP2

GACAATCATTGTTCCTCAAAGCACCAAAACAAATTGTCAAAGTATTAGAAAAGATTAGAT
AGGGAGAGGGTTTTGACAAACAATGATGTCAAGTGACCGGATCACCCTTAAAATTGGTCC
GCCTTTACTATTTTCTTTTCCCGGCGAAGTCCGCGGTTTCTATTTGGTATCTGCATTTCT
CCACCAGGTATTTTTCAATTCCATTGTGAGTGCCCACACAAGTGGCTCTGTTGTATATGG
CCTAGTCATACTTCTCAGTTCTCACTCTCACTCCGTAGAAAGAACCCTAGAATCAGAATT
CTCCGTCTATGTTGCAGAAGCAGCTACCTCCCCCCATTGAGGCCTCTTCCGATGAAGAGC
AACGACCTTCCTCCAAGCAACACACAGAAGAAGGAGTTTCCTCTTCAGAAGAAGCTTCTT

CCCAAGAAGAAGACGACGACGACCAACCTCCCACCCTTCCTCTCGCTTCCGCAAACCCCC
ACCCGAAACCGTCGTCCTCCGACTCCGACACCGACTTCGAGCCCACCAAAGTAAAACCCA
AGCCCACGGACCAGGCCCAGAAGCCCCAGCCCTCACCCGCGCCGCCCAAATGGGGATCCA
AGCGCCCTGCCCAGAACAACGCCCCTGCCACTGACCCGAAACGCGCGAAGAAGAAATTGA
CCAATTCTTCTTCCGCCGCTGCCGCCCACGAGACGGAGGAGAAGTCCGGTGGCGGCCAAG
CGAAGTTGTCTCAGAGACTCTTCAGCAAGGAAGACGAACTCGCCATTCTTAAGGGCATGG
CTGAGTTCATTTCGAAGACGGGCCAAGACCCTTACAAGTACGCCGATGCCTTCCAAAATT
TCGTTAAGAATTCGCTCCGCGTGGAGGCTTCGAGCAACCAGATCAAGGAGAAGATCCGAC
GGCTGAAGAAGAAGTTTGAAACTAAAGCACAGAGAGCGAAGAAATGGGAGGACCCTGAGT
TCTCCAAATTTCACGACCGGACTGTGTTTGAATTATCAAAGAAGGTTTGGGGAGAAGGAG
CCAATGGGCTGGTGGAGAAGCCAAAACCTAATAATGGAAAAAGAAAAACTGCCAAGACTC
CAAAGAAGGATGCTACTAGCAGGAATGTAGTAGCAAAATCTGAAACTACAACACTACTGG
AGTCGATGGAGTTGGAGGAGTGTGGGAATGTAAATTTGCTTTACCGCGAAGTATCTGGTT
TCAAAGAACTGAATGAGGATGAGATGAAGAGGGGATTGGCGTTAATTGGAGAATCTAAGA
GGAAAGAGTTGGAGGGCAAGTGGAGGAAATTGCGACTTGCTGAGATGGAACTGGTTGCGA
ATCGCTCACTACTTATTGGGGAGCAGATTAAGTTGATATTTGAAGCACTTCAGTAATCCA
ACAACTAGGGTTTATTGTGTTAGGTATCACTTTTTGCCTTTTCGGTACTGCCTGCCATAT
GTATTGTGGATCATTGTAATTTTGTGTCTATGGTATTGATATAGTCTTTTTTATTAAGTA
GGCAGTGTTGTTTAGTTGTTGGCCTTGTGAAAATGTGCCGCATAGTCGTCCATTTCAGGT
TCTCTTTTTCAATTAAGATGTGCCATCATTAACTCCAGAAAATGCCGAAATTAAACACTT
ACCATTTTGTGTAGATATATGATGGACACATCATGGTTATGTGTACGTTTCAGATCACCG
CTACTTACGTGAAAGGCAATATTGCTCTGACTTAGAAAGTTTTTAGTCTTGATTAATTGT
GCATTGTGGGTTCAATGGTGTTAAGTAACTGTTATAGTGCCATAACATTATAACCTGTGG
TTTTTGATCCCTCCACCTTAGGCAATTTGTGAAGGGAGGGCCACTATGATGGTGCCATAG
GCTACAATGGTGTGTTTATATGGCGAAATTTCAGCATTCCACCACTGATAACCTCAAGTG
GGTTTTGTCTTAATCATCATCCTTGGTGTTTATAAGGGAGGCCATTTGATGCACTTGGTA
GCATAGCCTTTTCAAATCTTAGTAACAAGTAGCAAAATTTATTAACCATTTTAATAAATA
ATGGAGAAAATATGTGGTGCCAGTTATGGCAATATGTTCAATCAGGCAAATCCTTATTTG
TTAACTGTGGGTTTGCAATTTGGGATGGCTGGAACATTCATCATCACCAAGGCCTGGCTG
GGGATCATGGAATGAACTGCCTTGTGTTAACTGTCTATTGCAATGACATTGCTGCGTTTG
CCCTTGCTCCTTTTGTGCTTATAACTTTGAAAAGTGCAACTCATTTTGTGATACATCTAA
AATTACTAATGCATATGCTCTTTACAAACAAGTGTAGTTTTCAGCACATAATCCATATGT
CATGTTTGTTAACTATTCTTTTACAACTTTAAAATTTTGGGACAAGGTTTCTCTTAACAT
AATTTATTCGGAATGGAACTTCTGTAGTAAAGTAGTCTATGGGCAAATTTCATTGAACTC
CTTGAGATCTGTTTAAATATCACCAACACTTCCTTTATTTTCTTAAAATCTCTAACCTTT
CTCACATTGGCACTGTTTAGCTACTGTTTTAAAAGGAAAAGACTTTTCTTTTAGCTATTT
GTTTTTGCACTCTTTTTGGCAATCTCACTTGCTATATTGTTCTGAAATATGCTAGTTAGG
CAACTCCATCCCCTAAATAAGATGTGTGCACCAAAAAATGACTATGCTGGGAAACTTGGA
CTATTTTCACAAGTCCGAAACACTAATCAGAGGCCTCACACTTCTTACTTCTTCAGAAAT
CCCAAAAGTAAATAGAAGGGAAGTGCTCCAAAAAGTATATGTTTAAGATGCTGAAACACA
TTCCAAGTTTGCAAAGCTTATTTTGCAATTGTAAAATTTAAGCTATAGATTTAAAGACTT
GCTCTTAGGATAGCAAGTATCTCTTTGCATATATACCCCACTTGAAAATGCCCAAGAACC
TATTCTCTGGGAGCCGAATAAGGTAGATAATACAGAGTCCAAATGTTGGTAAGTTTTGTC
TGTCCAAATACTCTTTTTTTGTCTCATGGAAGGGGAAGGTTACACTTTGGTACATCTCTA

TAGTCTTCTGATTAATTGGGTGAGTCTTTTCTAGAGATTATTGTCCCCCATCACCATGTG
CTAGTTTTTCTAGAAGAATCTTTTGGCATCCTTGTGTTCTATTTCAATTTTTAAGTATAT
AACTTTTAACTTTTATGCTTTTAGATTTAAATATGTTTTATTTTTAATTATTATCGTAGC
TTACTATGCCTGTCACAGATGCAATCATTAAATTATTATGCTTTAACTGGGAATGCTCAT
TTTTGACCTAGTGTTGATCATGCAGTAGTAAAATGCTTGTTTGTTTTTGAAGAGTTTTGC
TTTGTTTGCTTGTGAATTGACTACCTATGAAATTTCTTGTTATGGTTCTGCCATCATTTT
GAACAAACATCATTTTACCTTTGCTCTGTGATTCTTTTGTGTCTTGCTATGTTCTGTAAC
TGGTATTTACATCGTGCAGTCAACTTTTTGTGTCTCCCTCCTTTGTAAGGAGCTTCAATG
TTTGCTGTCATTTGAAAGGGGCTCAATTGGTATGCTACAAAGGTAGATCTTTACAAGGTC
AATTATTGTCTAAATACTGTAATATGTTATAGCAAATCATGGCCA
>GmGeBP3
ATGGAATCCGATCTAAACGACGCCGTTTTCCCCGAAGAAGATCTCGACGACGACGACGAC
GATGAAACTCCAGAGGACGAAGAGGACTACGACGACGAAACCGAACCCCCTCCCTTCGTC
CTCGCCGTCGTAGCAGTCGCGCCGCCCGCTTCCACCGCCTCCGAAACACTCGACACCACG
TTGATCCCGATCTCCGCCGTCGCCGATTCCTCACTGAAACCACTGCACCCGGAGCTAATC
GAGGAGAAAAAAGCGCTGGACGATTCGCGGCGACTGTTCCAGCGTCTGTGGACGGACGAG
GACGAGATCGGGCTCTTGCAGGGGTTTCTGGACTACACGGCGCAGCGAGGATCCTCTCAC
CACAGCGATACCGCCTTGTTCTACGACCAAATCAAGTCGAAGCTCCAACTCGGCTTCAAC
AAGAACCAGCTCGTCGAGAAGCTCCGAAGGCTGAAGAAGAAGTACCGCAACGTCGTCACC
AAAATCAGCTCCGGCAAGGATGTCTCCTTCAAGAGCCCTCACGACAAAGCCACCTTCGAA
ATCTCACGCAGGATATGGAGCAACACCGCTCCAATAAGCGGTCCCGTCGAAGACGACGAC
GAAATCAACCCTAACCCTAATTTCGGCAATTCCGCGAAGACACCTATCTCGCGGAAGCGG
TCGCGGCCTCAGAAGCGCGAGTTGAACGACGGTTCAACATTGAATAGAGATAATAATTGT
ATTGGCAATAATAATAATAATAGCAATAGTAATAATAATAATAATGAGAATTGTAATGGT
AGGCATAACTTACAGGGTTTGATTGAAGAGACGGTTAAGAGTTGCGTGTCGCCGGTGTTG
AAGGAGTTGGTGTGTGGTACCGGTGGCATGGAATTGGGAAGAGGGTTTGGAGTTGGAGGA
GGGCTTGGAGTTGGAGGAGGGCTTGCATTGAATTCCTTGCAAACTCAAATGACAATGCCA
ATGACTTTATTGAATTTGAGGATTGGGGAAACAACGATGGATGAGAAGTGGAGGAAGCAA
CAGATTTTGGAGCTGGAAGTGTATTCCAAGCGTTTGGAATTGGTGCAGGATGAGATCAAG
GTTGCTCTCGAGGAGTTGCGATCAGCTGGAGGAGTATAACCCAAAATAAAACTGAGCTTT
CAGGTGTTTACAGTACTCATAATCCACTAATGGTTGATAGGGGTAGCTAGACAGTTTGTA
ATTTTTTTTTTATAGTTTAGCTTTTTTTCAGTATAGCTTTTGATTATTGCATACAGTATT
TGTTCAATAAAACGTTCTTATTTTTGTTTATTTGACTTTTGTAAAATTTCTTCTTGGCTA
ATTAATAATTTACATGTACAGATAGAGTCAAGGAATTGGCGCAAGATGAACGGCCTTGTA
TTTTGATGTGTTGTTTAGTATGCAATTTTGGGTGAATTTTCTTACGAAATTACATGTATC
CCGCAGGGCTTTTGGGTTTTGTACTTTCTGAGCTGTGTTTAGGATAGTGGTATGCAATGG
CATATTCCAGATCCTGAAACTTCTTTTGGATGGTCATTCTCGTATCTCTCCTGGTATCTC
ATCAGTGTGATCATTTGTGGATTTTGCTGTATGGAAGTACTGAAAGTTGAAGATTTGAGA
TTGGAATGAAGAGCAATTTCTGTTGTCTGTTATAATGCCATTCGGTAGTTATTACTACTA
TTTGATTCAGGTAAAGTTGAAATGTGCACTTTATTTCTGCCTTCCAATGATTTGGATCCT
CTGATACTCGGAAAGAGGCTGGGGCATGGGTTGAAACTTGAAAGACATTGTTTATTCGGA
TTAATTCAATTATTAGTTTATCCGATATCACTTTTCA
>GmGeBP4
ATGCACAACTGCCATAAACGAACAAACGGCTACCTTGACACTCTAATATGCGAAAAAAGG

AAATGTGATATCTCCTCAACAATGCTTTCCCGGTTGGTTTCTTGGATCTTGTCGCCCTCC
TCTTCTGAAGAAGAAGAAGAAATCCTTGACATCATCAAAGATAATGACATTAACCATGAA
AACGACCAAAAGTTCAACGTAGAAGATGACAAGAACGACCATTTTCTTAACTCATGCGAT
GTGGACGACACCATTCCCATTGCACTTGCCGTTCCCAATGCCTCCCCCGCCGTGACAGTG
GCCTTCCCGGCCAATGACGAAAGAAACACCGTCCCAACCACCGCCACTGTGGCCACTATA
GTAACACGCTCAAAAGGGCAGCGCAACGCCAAGTATTCCGGAATGGTGAGACAATACCAA
AGGCTGCGGACGAAGGAGGATGAGATGGAACTGTTGAAGGGATACCTTGATTACGTCAAG
CAGCATAGAAAGGAAACCACCACCCTTCTTTACGTCGTAGTTTCATGTATGATCACGTAA
GGCCCAAACTAAACATCGATTTCAACAGAAACCAGCTTGTTGAGAAACTGCGTAGGTTAA
AGAGAAAACACAAGTTGGCTTTGGAAAAAGGCAAGGACAAGGAGGTTCCCTTTAGGAACC
CCCAGGAGCAGGCCATTTTTGAAATTTCCCACAAGATTTGGGCCAATGACACAGATAATA
TAATAGTCCAAGATGCCTTGGATGGTGATGAATCAGGACACACTCCTGAAAGTCATGATC
ATGTTGGCAACGTCAAGGTGAAGATTGAACAAGTTGACAACAGTGATGAAATAGGCAATA
GAGTGCCAAAGCGGTTGCGGCTAGATGATGCAGACGATATGAACAGAACAAATGACCAGA
ACAATGGTGATAGCATACAAGGCTTCATTGAGGACACCATGAGGTCCTGTTTCTCACCAT
TGCTGAAGGAAGTGTTGGATGAAGCACAGGAAGAGTCACTTCCTGAGTTGGAGGCAATCC
CAATGCCGCTATGCTCCGGGGAAGTGGACCATGAACAATGGAGAAAACGGAGGATTTTGG
AGTTGGAGGTGTATGTGAAGCGGTTGGAGTTGTTGCAGAATCAGATCAAGTCTAGATTGG
AAGAGTTGCGATCTAGCTGA
>GmGeBP5
CCTATCACTCCTCTACCAGGATTGTTTTTTTTTTTGGTTATATATTCCCAAGTCTGCTGT
CACTACCCACTACCTAAGCTCTTCTCACTCACTCAGTTAGTTGTTCCGAAAACCCTAATT
ATAATTAAATTTAATTTCACCATGGCACAGAAGCAAAAGCTGCGCCCTTCTCCTCTCGAC
GAGCCACCCACTGCTTCCTCTTCCGATTCCGAGGAAGAAGAGCCACAACAACAACAACCA
TCCTCCCAGAAGAACAAAGAAGAGGAAGACGAAGAAGTTTCCTCCGGAGAAGAAGAAGAA
GAAGACGAAGAAGAAGAAGAAGCTGCTTCCTCCGAAGAAGAGGAAGAAGACGAAGATCTC
CCACCACCACCAGTTTCCAAAAACCCTCCACCCCCTCCCGCAAACCCTCAGCCCCAACAC
TCTTCCTCCGAATCCGAAACCGAATCAGGATCCGAGACCGAATCCGAACCTGACCCCACT
CCCGTCAAGGTCAAGCCTTTAGCTTCCAAGCCCATGGACCAGGCCCAAAAGCCCAAGGCC
CAGCCCTCCCCGGCGCCACCCCCAAAATTGACACTCAAGCGCCCCGCCGAGAACAACAAC
AACAACGCCCGCGTCGCCGACTCCAAACGCGCCAAGAAGAAAGCCACCGAATCTTCTTCT
GCCGCCAACTCCGCCGCGGCCGCTGCTTCTGACGACGAGATGGAGGAGGACGGGAAGAAG
TCCGGCGATAACTCGAAGAAGTTTCAGAGACTGTGGAGCGAGGAGGACGAGCTCGCCATT
GTGAAGGGCGTGGTTGAGTTCACTTCGAAAACAGGGCTGGACCCTCTTAAGTTTCCCAAT
ACCAACGCTTTTCACGATTTCGTGAAGAAGTCGCTTCACGTGGAAGTTTCCTGCAACCAG
CTGAAGGAGAAGGTCCGAAGGCTCAAGAAGAAGTTTGAGACCCAGGCTGGAAAAGGGAAG
AACGGAGAGGCCCCTAAGTTTTCCAAACCGCACGATCAGAAATTCTTTGAATTGTCCAAA
AAGGTTTGGGGACGTGAGGTTACTGCTGGAGCTAATGGGGGCCCCGTGGAGAAGCCCAAG
TCTAATGGGAGTGCTGTCAAGAGTCCGAAGAAGAAGGAAAGCGGTAGCAGGAATGTGGCT
TCTGCTAAGAAACCGAAGCCTGAATCAAAACCGGAGCCGGTTCCAGTGCTGTCCTTGGAG
TATAAGGATTCTGAAAAGATGCAGATTAATCAAAAGCCTGATGGTGGTGATGCAAGTTTG
TTTTTGCGCGAATTGGCTCGATCCAAAGAGGGTGCGAGTATTTGTAAGCTGGATGAGGAT
GATGTGAAGAGGGGGTTGGAGTTGATTGGAGAGTCAAAGAGGGCAGAGTTGAGGGGGAAG
TGGAAGAAATTACATCTTGCTGAGATGGAACTGTTTGCGAATCGCTCAGAACTGATAGGG

GAGCAGACTAAGTTGATACTTGAGGCGCTTCAGGCATCCGATCATTAGGATTTCTCAATT
TTGTTAGGCGGCCTTTTTGGTACTCTGTGCCGTATATTTTGTGACCTATCCTCTATGCAT
TGTAATCATGTGGTTATGGTACGTACAGTCTTTTAATTCAGTGGCAGTAGCAGTGTTTAG
TTATTGACTTTATGAAAATATCTTGCTTCAGAAGTTTCAAGGAATTTTGTTTCAAATGAT
TCTCTATAATTTATAACTTATTTTGAATTGGAACTTTTGTGGTGGTATAATCTTTTGGGT
TTTTAGTCTACTTCACAAGTGATTGTTAATTGACTATGAGATATCTTATTTTTTTGTTGG
TTGTGTC

>GmGeBP6

GTTGGCCTCTACTATATATATATATATTCCCAAGGCTTCTGTCACTACCTAATAATCTCT
TCTGAATGTGCTCGTTGTTTAGAAAACCCTAATCTGATTCAATTCCAGCATGGCTCAAAA
ACAAAAGCTGCGCCCTTCTCCTCTGGACGAGCCACCCACCGCTTCCTCTTCCGATTCCGA
GGAAGAAGAGGAGCAACAACAACAACAACCATCCTCTCAGCAACACGAAGAAGAGGAAGA
AGAAGTTTCCTCCGGAGAAGAAGAGGAAGCTTCCTCCGAAGAAGAGGAAGACGAAAATCT
CCCCCCACCACCCATCTCCAAAAACCCTCCACCCCCTCCTCCCTCAAACCCTCAGCCCCA
ACCCACTTCCTCCGAATCCGAAACCGAATCGGGTTCCGAAACCGAATCAGAACCCCACCC
CACTCCCGTTAAGGTCAAACCCCTAGCCTCCAAGCCCATGGACCAGGCCCAAAAGCCCAA
AGCTCAACCCTCCCCGGCGCCGCCGCCGCCAAAATCGGCATCCAAGCGCCCCGCCGAGAA
CAACAACAACAACGCCCGTGTCGCCGACCCCAAACGCGCGAAGAAAAAAGCCACCGAATC
TTCTTCTGCCGCCGCCATCTCCGACGACGAGATGGAGGAGGACGGGAAGAAGTCCGGCGA
TAACTCGAAGAAGTTTCAGAGACTGTGGAGCGAGGAGGACGAACTCGCCATTCTGAAGGG
CGTGGTTGAGTTCACATCGAAAACAGGGCTGGACCCTCTTAAGTTCCCCAATGCCAACGC
TTTCCACGATTTCATGAAGAAGTCGCTTCACGTGGAATTTTCCAGCAACCAGCTGAAGGA
GAAGCTCCGAAGGCTCAAGAAGAAGTTTGAGACCCAGGCAGGAAAAGGGAAGAATGGAGA
TGCCCCTAAGTTTTCCAAACCGCACGATCAGAAATTCTTTGAATTGTCCAAAAAGGCTTG
GGGAAGTGAGGATGGTGGTGTAGCCAATGGCTCCGTGGAGAAGCCCAAGTCCAATGGGAA
TGCTGCCAAGAGTCCGAATCCGAAGAAGAAGGAATCCGGTAGCAGGAATGTGGCTTCTGC
TAAGAAACCGAAGCCTGAAACAAATCCGGAGCCGGCGCCGGTGCCGTCCTTGGAGTTTAA
GGAGTCTGAAAGGATGGAGATTGATCAAAAGCCTGATGGTGGTGATGCATGTTTGTTTTT
GCGCGAATTGGTTCGATACAAAGAGGGTGCCAATGTTTCTAGGCTGGATGAGGATGATGT
GAAGAGGGGGTTGGAGTTGATTGAGGAATCAAAGAGGGCAGAGTTGAGGGGGAAGTGGAA
GAAATTACATCATGCTGAGATGGAACTGTTTGCGAATCGCTCAGAACTGATTGGGGAGCA
GACTAAGTTGATACTCGAGGCGCTTCGGTCATCCAATCATTAGGATTCTCAATTTTGTTT
GGCGGCCTTTTCGGTACTCTGGGCCATATATTTTGTGATCTATCCTCTATGCATTGTAAC
CTTGTGGTTATGGTATAGTCTTTTAATTCAGTAGCAGTAGCAGTGTTTAGTTATTGACAT
TGTCAAAATATCTTGCTTCAGAAGTTCCTAGGAATTTTGTGACAAATGTTTCTCTATAAT
TTATAATTTATTTGGAATTGGAACTTTTGTGGTAGTATACCCTTTTGGGTTTTTATTCTA
CTTCACAAGTGATTGGTAGTAATATCTTACTTAGGTATCTAATTTATTAGTTAAATGATT
TGTTGTCCTTCTTGTTATTTATTTTCTTTCGACCATTATGGTGATCATTGTTTCATTGTA
CATCAAGGAATTTTAGTTTGAAGGATTCATATTTTGGTTATTTTTGTGATTACATCCAAT
GATGTATGTGATATGGGAATTGGTGTGGTCACTGAGATGGTAGTTAATTTCCCCTATCAT
GAAGTCTGATCATTTCTAAATTACATTTGATGGCCAAGGAAAATCCTCATTTTGTGAGAA
TGCTTTGATCAATGTGAGAATCTTGT

>GmGeBP7

TGGTCCACTAGGCTTACCTAAACCAAAACCAAAACCACCCTTCGGATTCCCATTACCAGC

AGCGAAGTGAGTGAGTCAGTCCAACTATAACCGAAAACCCGTGACTCAGCCCCACTTTTG
CTTTGCTTGGCCTAACCACTAACCAAACCAAACCTCTCAAGTCTCAATGGCTTCTCAGCA
ACACGACGCCGTTTTCCGCGAAGAAGACATGGACGACGACGACGACGAGTCTCAGGAGGA
CGGAGACTACGAAGAAGAAGACGACGACGTTTTGGCCGACGACGAAGAAAACGAGCCCTC
TCCCTCCACGGCTCTTGCCGTCACCGTCGCCGTCCCCGGTTCCTCCGTCTCCAACGGCGG
TGCCGCTCCAATTTCGACGCCCACCGCCACCACCATCGTCGTCGCAGACTCCTCCGATCC
GAAGCGGCGGCGCCTCGAGCCAGTCGAGGAGAAAAAGCCGCCGCCGACGCCGGACGACTC
GCGGCGGCTGTTTCAGCGGCTGTGGACCGACGAGGACGAGATCGAGCTCTTGCAAGGCTT
CCTAGACTACACCTCGCAGCGAGGATCCTCGCACCACAACGACACGGCTTTGTTCTACGA
CCAAATCAAGTCGAAGCTCCAACTCGATTTCAACAAGAACCAGCTCGTCGAGAAGATCCG
AAGGCTGAAGAAGAAGTACCGAAACGTCCTCAACAAGATTGGCTCCGGCAAGGAATTCTC
CTTCAAGAGCGCTCACGATCAAGCCACCTTCGAAATCTCGCGCAAGATCTGGAGCAACGT
GACTCCAATCGGCGACAATTCCTTGGACGACGACGAAATCAACCCTAACCGTAGCCCCAA
CCCTAACCTTAATTTTAGCCCTATAATTCTTAAGAATGAAATGATTTTCAGGAACCCCGC
GGAGAAGAAAACACCGAAACGCTCTCGGCCACGATCGGCGGTGAAAATCGAGCCAAATGA
CGGATCCGCGTCGAACAGAGATCATGATTGTATTAGCAATGCTACGCCTACTGCTACTGC
TACTGCTGCTGCTACTAACACTACTCCTACTGCAGCTACTACTAATGATAACTGTAACAG
TGGTTATGGAAATAACATACCGAGTATGATCGAGGAGACGGTGAGGAGTTGTTTGTCGCC
GGTGTTGAAGGAGTTGATGGCAGGGGCCATGGGAGGAGGAGCGTTTGGAGCGAGAGGGTT
TTCGTTGAACTTGAACCCTATGCCTTTGATGAATTGGAGTTTTGGCGGTGGGGAAATGGT
GGATGAGAAATGGAGGAAACAACAGATATTGGAGTTGGAAGTGTACTCAAAGCGGTTGGA
GCTGGTTCAGGATCAGATCAAGGCTGCTATGGAGGAGTTGCGGTCACACGGAGGAGGTTG
ATCAAATCTTTTAGTTTGTTTTTGGTTTTGTAGTGAGTGATTGGAGTTCTTCTTCCTCTT
CATCATCAATGGTTGGTTCATTCTCATAGCTTAGTGGTTAGCTAGTAGTAACTAGTAAGT
GGTAACTAGTGTTTTTCTTTTCGATTTTAGCTTTCGCTGATTCGGTGGAGCACTTGTCCT
TTAAAATAAAAATGTTTTTAGTACATTTTAGCCGTTTTTATGTTCCTCTAAATTTCAATG
GTCTCATACATGTACAGAACAGATTGAAGTAACTGGTGCGGAGTGATGGCTTTGTACCAT
TATATATGTTGTAAAAGATGCGAAAGTAGTGAGTTCTTAATTGTGCAACATTCTTCCCGA
GTACTTTGCTCTTAAAGTTTTCATGCAGTATTCTGTGTCTTCGGGGTTTCTTGCTATATC
TTTCGTATTTTTATGATTCAATGGATTGTAAAGATGGCATTTTCTAATCTGAAAGAGATT
CTGCGATGTTCTGTGTCAATTGTTGAATAACGAAACAATGAATGCTGTAAACCAGGGGTT
GTTGAAGAACCCCTCGATATTCATATATGCTGCAAGTGATTGGGGCTTGAAGCTCTTGGA
ATTTTGAATGC
>GmGeBP8
GAAATGTGGTATCTCCTCAACAATGCTTTCCCCGTTGGTTTCTTGGATCTTGTCTCCCTC
CTCTTCCTCCTCCTCTTCCGAAGAAGAAGAAGAAGAGATCCTTGACATTATCACAGATAA
TGACATTAACCATGAAAATGACCAAAAGCTCAATGTAGAAGATGACGAGTGTGATGTGGA
CAACACCATTCCCGTTGCACTTGCTGTTCCCAATGCCTCCCCCGCCGTGACAGTGGCCTT
CCCGGCCAATGACGAAAGAAACACCATCCCAGTCACCGCCACTGCCACCACTGTCGTAAC
ATGCTCAAAAGGGCAGCGCAACGCCAAGTATTCCGGAATGGTGAGACAATACCAAAGGCT
GTGGACGAAGGAGGATGAGATGGAACTGTTGAAGGGATACCTTGATTACGTCAAGCGGCA
TAGAAAGGAAACCACCACCCTCCAAAGCGTCGTAGCCTCGTTGTATGATCACGTAAGGCC
CAAACTGAACGTGAGTTTCAACAAAAATCAGCTTGTTGAGAAACTGCGTAGGTTAAAGAG
AAAACACAAGTTAGCTTTGGACAAAGACAAGGACAAGGAGGTTCCCTTTAGAAACCCCCA

GGAGCAGGCCATTTTTGAAATTTCCCACAAGATTTGGGGCATTGACACGGATAATATAAT
AGACCAAGATGCCTTGGATGGTTATGAATCAGGACACACTCCTGAAAGTCATGATCATGT
TGGCAACATCAAGGTGAAGATTGAACAACTTGACAACAATGATGAAATAGATAATAGAGT
GCCAAAGCGGTTGCGGCTAGATGATGCAGATGATGTGAACAAAACAAATGACCAAAACAA
TGGTGATAGCATACAAGGCTTCATTGAGGAGACCATGAGGTCATGTTTCCCACCATTGCT
GAAGGAAGTGTTGCATGATGCACATGAAGAGCCACTTCCTGAGTTGGAGCCAATCCCAAT
GCCACTATGCCCTGGGGAAGTGGACCATGAACAATGGAGAAAACGGAGGATTTTGGAGTT
GGAGGTGTATGTGAAGCGGTTGGAGTTGTTGCAGGATCAGATCAAGTCTAGATTGGAAGA
GTTGCGATCTAACTGAGGATGACATAAGTTTTATATTGCTCCCAATTGTGCAGAAACATC
ATGTGCCTTATTCTACACTAACAATTGCAATGAATAGTAGAATAGCTCTAGCAATTCTGT
AATGTTTTGTAGTAGACAAGAAATT
>GmGeBP9
ATTCTTATTGCCAGCGAGTGAGTCAAAAGAGACACTAACAAAACAAACCCCGTGAAAGCA
AAGCACCCCACCGCGCTCTTTGCCTAACCGACTCACTCCCATGGAATCCGATCTAAACGA
CGCCGTTTTTCCCGAAGAAGATCTCGACGACGACGACGAAACTCCAGAGGATGAAGAGGA
GGAGGAGGACGACGACGTCTTAGATGACGATGAAACCGAACCCCCTCCCTCCGTCATCGC
CGTCGCGCCGCCCGCTTCCGAAACGCTCGACACCGCGTTAATTCCGATCTCCTCCGTCGC
GGATTCCTCGCCGAAACCGCTGCGCACGGAGCTAATCGAGGAGAAAAAAGCGCTGGACGA
TTCGCGGCGACTGTTCCAGCGTCTGTGGACAGACGAGGACGAGATCGGGCTCTTGCAGGG
TTTTCTGGAGTACACGGCGCAGCGAGGATCCTCTCACCACAACGACACCGCCCTGTTCTA
CGACCAAATCAAGTCGAAGCTCCAACTCGGCTTCAACAAGAACCAGCTCGTCGAGAAGCT
CCGAAGGCTGAAGAAGAAGTACCGCAACGTCCTCAACAAAATCAGCTCCGGCAAGGAAGT
CTCTTTCAAGAGCCCTCACGACCGAGCCACCTTCGAAATCTCGCGCAGGATCTGGAGCAA
CACCGCTCCAATCACCGGTCCCGTCGAAGACGACGACGAAATCATCACTAACCCTAATTT
CGGCAATTCGGCGAAGATGCCTATTTCGCGGAAGCGGTCGCGGCCTCAGAAGCGCGAGTT
GAACGACGGTTCAACGTTGAATAGAGATAATAATTGCAATAGTAACAGTAATAATAATAA
TAATAATGAGAATTGTAATAGTAGGCTTAACTTACAGGGTTTGATTGAAGAGACGGTGAG
GAGTTGCGTGTCGCCGGTGTTGAAGGAGTTGGCGTGTGGTACTGGTGGCATGGGATTGGG
AAGAGGGTTCGCATTGAATCCCTTGCAAATGCCAATGCCAATGAGTTTAATGAATTTGGG
GATTGTTGGGGAAACGGCGATGGATGAGAAGTGGAGGAAGCAACAGATTCTGGAGCTGGA
AGTGTATTCCAAACGTTTGGAATTGGTGCAGAATGAGATCAAGGTTGCTCTCGAGGAATT
GCGTTCAGCTGGTGGAGGATGACCCAAAATAAAACTAAGCTTTTAGGTGTTTACAGTACT
TCATAAGGATTTGCTTGCTAATTTATTGATCCACTAATGGTTGATAGGGTAGCTAGACAG
TTTGTATTTTTTTCCCCTTTTTTTCTATAGTTTAGCTTTTTCCTTCTTTTTTTGCTTTTC
AGTATAGCTTTTGATTATTGCAAACAGTATTTGTTCAATAAAATGTTCTTATTTTTGTCT
TTTTGGCTTATGTAAAATTTTCTTCTTGGCTGATTAATAATTTACATGTACATGTACAGA
TTAAGTCAGGGAATTGGCGTAAGATGAAAGGCCTTGTATTTTGATGTATTGTAAAGTATG
AAATTGTCAATTGTGGGTGAATTTTCTTTCG
