# Supplementary material for: Genome-Wide Characterization and Expression Analysis of GeBP Family Genes in Soybean
Source: Plants (Basel). 2022 Jul 14;11(14):1848. doi: 10.3390/plants11141848 (PMC9318833; doi:10.3390/plants11141848)
Supplement: Supplementary file 1 [file plants-11-01848-s001.zip › Supplementary file 3.pdf]

>GmGeBP1
MASEQHDAVFREEDMDDDDDESQEDEEYEEDDDEENVPSPSTALAVTVAVPGSAVSNGGG
GSPISKPTATATTATIVLADSSDPKRRRLELIEEKKPPPPLDDSRRLFQRLWTDEDEIEL
LQGFLDYTSQRGSSHHNDTALFYDQIKSKLQLDFNKNQLVEKIRRLKKKYRNVLNKICSG
KEFSFKSAHDQATFEISRKIWSNVTPVGDNSLDDDEINPSRSPNPNLNFSPVILKNETIF
RNSTEKKTPKRSRPRSAVKIEPNDGSASNRDHNCISNTTPTATAAATNTTAAAAAGTNNN
NCNSGYGNNIPSLIEETVKSCLSPVLKELMAGAMGGGAFGGRGFSLNLNPMPFMNLSFGG
GEMVDEKWRKQQILELEVYSKRLELVQDQIKAAMEELRSHGGGGL
>GmGeBP2
MMSSDRITLKIGPPLLFSFPGEVRGFYLVSAFLHQKQLPPPIEASSDEEQRPSSKQHTEE
GVSSSEEASSQEEDDDDQPPTLPLASANPHPKPSSSDSDTDFEPTKVKPKPTDQAQKPQP
SPAPPKWGSKRPAQNNAPATDPKRAKKKLTNSSSAAAAHETEEKSGGGQAKLSQRLFSKE
DELAILKGMAEFISKTGQDPYKYADAFQNFVKNSLRVEASSNQIKEKIRRLKKKFETKAQ
RAKKWEDPEFSKFHDRTVFELSKKVWGEGANGLVEKPKPNNGKRKTAKTPKKDATSRNVV
AKSETTTLLESMELEECGNVNLLYREVSGFKELNEDEMKRGLALIGESKRKELEGKWRKL
RLAEMELVANRSLLIGEQIKLIFEALQ
>GmGeBP3
MESDLNDAVFPEEDLDDDDDDETPEDEEDYDDETEPPPFVLAVVAVAPPASTASETLDTT
LIPISAVADSSLKPLHPELIEEKKALDDSRRLFQRLWTDEDEIGLLQGFLDYTAQRGSSH
HSDTALFYDQIKSKLQLGFNKNQLVEKLRRLKKKYRNVVTKISSGKDVSFKSPHDKATFE
ISRRIWSNTAPISGPVEDDDEINPNPNFGNSAKTPISRKRSRPQKRELNDGSTLNRDNNC
IGNNNNNSNSNNNNNENCNGRHNLQGLIEETVKSCVSPVLKELVCGTGGMELGRGFGVGG
GLGVGGGLALNSLQTQMTMPMTLLNLRIGETTMDEKWRKQQILELEVYSKRLELVQDEIK
VALEELRSAGGV
>GmGeBP4
MHNCHKRTNGYLDTLICEKRKCDISSTMLSRLVSWILSPSSSEEEEEILDIIKDNDINHE
NDQKFNVEDDKNDHFLNSCDVDDTIPIALAVPNASPAVTVAFPANDERNTVPTTATVATI
VTRSKGQRNAKYSGMVRQYQRLRTKEDEMELLKGYLDYVKQHRKETTTLLYVVVSCMITN
QLVEKLRRLKRKHKLALEKGKDKEVPFRNPQEQAIFEISHKIWANDTDNIIVQDALDGDE
SGHTPESHDHVGNVKVKIEQVDNSDEIGNRVPKRLRLDDADDMNRTNDQNNGDSIQGFIE
DTMRSCFSPLLKEVLDEAQEESLPELEAIPMPLCSGEVDHEQWRKRRILELEVYVKRLEL
LQNQIKSRLEELRSS
>GmGeBP5
MAQKQKLRPSPLDEPPTASSSDSEEEEPQQQQPSSQKNKEEEDEEVSSGEEEEEDEEEEE
AASSEEEEEDEDLPPPPVSKNPPPPPANPQPQHSSSESETESGSETESEPDPTPVKVKPL
ASKPMDQAQKPKAQPSPAPPPKLTLKRPAENNNNNARVADSKRAKKKATESSSAANSAAA
AASDDEMEEDGKKSGDNSKKFQRLWSEEDELAIVKGVVEFTSKTGLDPLKFPNTNAFHDF
VKKSLHVEVSCNQLKEKVRRLKKKFETQAGKGKNGEAPKFSKPHDQKFFELSKKVWGREV
TAGANGGPVEKPKSNGSAVKSPKKKESGSRNVASAKKPKPESKPEPVPVLSLEYKDSEKM
QINQKPDGGDASLFLRELARSKEGASICKLDEDDVKRGLELIGESKRAELRGKWKKLHLA
EMELFANRSELIGEQTKLILEALQASDH
>GmGeBP6
MAQKQKLRPSPLDEPPTASSSDSEEEEEQQQQQPSSQQHEEEEEEVSSGEEEEASSEEEE
DENLPPPPISKNPPPPPPSNPQPQPTSSESETESGSETESEPHPTPVKVKPLASKPMDQA

QKPKAQPSPAPPPPKSASKRPAENNNNNARVADPKRAKKKATESSSAAAISDDEMEEDGK
KSGDNSKKFQRLWSEEDELAILKGVVEFTSKTGLDPLKFPNANAFHDFMKKSLHVEFSSN
QLKEKLRRLKKKFETQAGKGKNGDAPKFSKPHDQKFFELSKKAWGSEDGGVANGSVEKPK
SNGNAAKSPNPKKKESGSRNVASAKKPKPETNPEPAPVPSLEFKESERMEIDQKPDGGDA
CLFLRELVRYKEGANVSRLDEDDVKRGLELIEESKRAELRGKWKKLHHAEMELFANRSEL
IGEQTKLILEALRSSNH

>GmGeBP7

MASQQHDAVFREEDMDDDDDESQEDGDYEEEDDDVLADDEENEPSPSTALAVTVAVPGSS
VSNGGAAPISTPTATTIVVADSSDPKRRRLEPVEEKKPPPTPDDSRRLFQRLWTDEDEIE
LLQGFLDYTSQRGSSHHNDTALFYDQIKSKLQLDFNKNQLVEKIRRLKKKYRNVLNKIGS
GKEFSFKSAHDQATFEISRKIWSNVTPIGDNSLDDDEINPNRSPNPNLNFSPIILKNEMI
FRNPAEKKTPKRSRPRSAVKIEPNDGSASNRDHDCISNATPTATATAAATNTTPTAATTN
DNCNSGYGNNIPSMIEETVRSCLSPVLKELMAGAMGGGAFGARGFSLNLNPMPLMNWSFG
GGEMVDEKWRKQQILELEVYSKRLELVQDQIKAAMEELRSHGGG

>GmGeBP8

MLSPLVSWILSPSSSSSSSEEEEEEILDIITDNDINHENDQKLNVEDDECDVDNTIPVAL
AVPNASPAVTVAFPANDERNTIPVTATATTVVTCSKGQRNAKYSGMVRQYQRLWTKEDEM
ELLKGYLDYVKRHRKETTTLQSVVASLYDHVRPKLNVSFNKNQLVEKLRRLKRKHKLALD
KDKDKEVPFRNPQEQAIFEISHKIWGIDTDNIIDQDALDGYESGHTPESHDHVGNIKVKI
EQLDNNDEIDNRVPKRLRLDDADDVNKTNDQNNGDSIQGFIEETMRSCFPPLLKEVLHDA
HEEPLPELEPIPMPLCPGEVDHEQWRKRRILELEVYVKRLELLQDQIKSRLEELRSN

>GmGeBP9

MESDLNDAVFPEEDLDDDDETPEDEEEEEDDDVLDDDETEPPPSVIAVAPPASETLDTAL
IPISSVADSSPKPLRTELIEEKKALDDSRRLFQRLWTDEDEIGLLQGFLEYTAQRGSSHH
NDTALFYDQIKSKLQLGFNKNQLVEKLRRLKKKYRNVLNKISSGKEVSFKSPHDRATFEI
SRRIWSNTAPITGPVEDDDEIITNPNFGNSAKMPISRKRSRPQKRELNDGSTLNRDNNCN
SNSNNNNNNENCNSRLNLQGLIEETVRSCVSPVLKELACGTGGMGLGRGFALNPLQMPMP
MSLMNLGIVGETAMDEKWRKQQILELEVYSKRLELVQNEIKVALEELRSAGGG

>MtGeBP01

MLSPLVSWILSTSSSSSSSSEEEEEESLDINNDDSDNEKFDNDNVEDQMDNVDDDEKDHF
LCPYNVDEDQMDNIPIALAVSDASPVVTVAMAAPADDTNAATNTATTSATTTITTRSKRQ
YNKKYSDGVKQYQRLWTKQDEIELLKGYLDYIKQQGRANTTIQSGVASFYDQVIPKFSVE
FNRNQLVEKLRRLKRKHKMTLDKGKEVQISFKIPQEQAIFEISHKIWGNDTDHDVLDVDD
SRPVPESPDLIENIKLKAEDVDNCEETDKRAHKRVRLTTDDVNSKNYQSNSDATSLRGFI
EETMKSCFSPLLKELLDDVPVEPPGALPMLLSTGEVRDEKWLKRRILELEVYLNRLELLQ
GQIKARLEELKSSLD

>MtGeBP02

MSTFATTAAIPTTIILALPNTAATANVNEDEITILQGFLDYNVNRGSSYHNDIGSFYDQI
KFRRLKKNHCMVLHKFDFGKDFAFKRAHYQATFEISHKI

>MtGeBP03

MAFSRNGVVIPTHDEHADIEEEDDDDLEEEDNYFVQPNTDHHYQTEQSPSTFATAAVPTS
VILALPHASASATTIDGSPEPKREPMEEKTSDSRKLFQRIWTEEDEITILQGFLDYNANR
ESSYHNDTGSFYDHMKGKIQLDFTKSQLVDKLRRLKKKYRTALQKFDSGKDFGFKSTHDQ
AAFEISHKIWNINGNSTPIGVPVEEDDEIIPNPNPNSSHLAEKKAVQSRKRSRAAEEERH

ELNDSKDNHQNNSGNTNSSNVDENREKSNGNHVHNVPGLIEETVKSCLTPVLKELSNATM
SGSPFGFGGRGFGAGGFSMNTVPLGFLNLGSGEKAVDDKWRKQQILELEVYSKRLELVQD
EVKAAIEELRSNAGAGGAGGSGNNTNQ

>MtGeBP04

MAPKPKKRLALVDDPPTASSSDEEQPPSTQKPADKVVNEQDEDSSSEEEEGDEEDGSSSE
DEEEEDNQTQQTPSKDSKPASKNPPPSTPISNPKPAESESGSESGSESGTESDSEPEHKS
EPTPPPNPKVKPLASKPMKTQTQAQAQSTPVPIKSGTKRVAESSTGNDSKRSKKKTTTAG
GGSDDENEVEEDAKLTGEDSKKNFQRVFSEEDELVILKGLGDFVAKTGKDPMKETAAFHS
FVKKLLKADANAEQLKRKVRGLKKKFESSDNFTKPHDKRAFDLFKVWSKNASKEAEENGK
SNEKIAKSAKKEAPVKNGGSAKKELAIVTKNVSGKVESGVGKSFILSELVKFDEAAGIAV
LDKDAAESGLELMRESDRVELEERWKKLQAAEMDVFLNRAEVVREQTRLAMQELKKKSST

>MtGeBP05

MAKKNSATAAVKDDPPPQEDKIEQHDENDEETSSEAGSSSEGGEEEEEESSEEEAPKTTPP
PKTNPKNNSVEDEDEEETDTDEDEPPPKTKPIEQTLKSGTKPSIAPARSGTKRPAENNDT
KQSNKKKTTEEKNKKKEKEPEEDDNSNNKKASFQRVFTEDDEVAILQGLVDFTAKSGNDP
TKHLSAFYQIVKKSVHFKVTLDQLRDKVRRLRLKYENKIKSKKTPTFSKPVEETMFELSK
KIWGGGKVDVNEGEENEKVNGKPAKKELAVKKAAAARTTKKLVMEPDSPLVVLKVDSGNG
KEDTGLSLVSNETIHFEPVIDVVKRGVESIEESKRGELERLMNKYKIAEMELFMQKTELA
KETATKIRDALNSSSSH

>MtGeBP06

MASQQHDTVFPEEDIDDEEDEFHEYEEEDEEDDDVLVDDEENDLPSASASISVPSVTVAV
PGGALRPPILTPTATTIIFADSTFSSDSKRQRLDQIEEKKPLLVEDSRKLFQRLWTDEDE
MELLQGFLDYTTQRGSSHHNDTAQFYDQIKSKLQLDFNKNQLVEKIRRLKKKYRNVVSKM
SSGKEFVFKTAHDQATFEISRKIWNNVAPIAGDIAIDDDEINLNPISNPNLNFTPVILKS
EVNSTTRKRSRPRSVSIRSFEKRESNDAVASNRDRDYLNINLNNPNPNPYPNHNHHPNPN
PNAYTTTATVNPAITPTITTPNVNTNAAIHANNYSNNCSTAGNGVAGVIEETVRSCLSPF
KELMAGAMGGGLFASRGSFAMNPLPFQMMNLGFGGGELADEKWRKQQILELEVYSKRLEL
VQDQIKTSLDELRSVGGSSQL

>AtGeBP01

MSRRFNPLEDPPSASSSDEEGKEIARNSSSDDEDDVSSENPSPLKTTLDAVSDSESGSDE
ETDSDSELEKKKDQVVTNPVDVKRAKKVKTSEKSGAKRSLEVDEAAVSMDVKRAKKVSGE
EEKKKSGGGGEETKKTYFQRLWTEDDEIVVLQGLIDDKKDTGVSNTNKVYELVKKSISFD
VSKNQLMEKLRALKKKYENNLGKAKDGVEPTFVKPHDRKAFELSKLVWGGIRMALASGMK
SNEKSKKSSKFESVKHELDSSLPNSKNNCEDEVMDEGEVSFTKSSLVRSIVGLGMDELTA
QQGLSKLASKDMKRFYEQWKAMQAREFEFFLQKHGFLFEVLSKISEAFGSNA

>AtGeBP02

MNKKLLNPLEDPPTASSSEDVDEEISSGEDEKEHISNSSSSEEENELKDLSTQTLNSPST
EAPTLDSGSETNSDSDKPIVLTSQKKKEGTDSSGTKRASEGTSSKDIKRAKKVSGDDDNK
KFQSLWTKEDEISLLQGMIDFKAETGTSAHDDMNGFFDIAKRYISFDVSKIQFGDKIRGL
KKKYFGVRKKKGLDLDHDKKCLGLAKSIWGLDGKEVVVLGGDSETSNWFEKSFLVRVVAR
LGVDECIVKWKWSKVSKETKKRIEEKMKMVEAKELELLSQKIDVLKEVASVIAETI

>AtGeBP03

MKRITKLLASHSLKSLMAKKNKRSQQKNKCLKPEKDPSTVKRLLEDPPRKKRYLQPLENP
PTESYGDDEKMETEEEGEKYLGKNLPASAFIIVAAERHDSDSYSETELEKKICGSVVVAK

MNDDKMVSTDTKKKYFQRILSDDDKIVLLQGMVDFQNDKGTISYDDMTGFIDTVKNIISF
QANSRQFTTKIRRLKDKFVQKRNKGVDENSLANVEIYEI
>AtGeBP04
MTKKLNPLEDPPTATSSDEDDVETSEAGEASDDSSSSEEDVPIKIRIKSPSATTAAAPPA
KSTAVSTAADSDSGSETETDSDSESTNPPNSGSGKTIALNTVNLKKKEDPTSSSATLALP
AMKSGTKRPASEAAATTSTKRVKKDEESVKKPGGFQRLWSEEDEILVLQGMIDFKADTGK
SPYVDTNAFYDFLKKSISFEVSKNQFMDKIRSLRKKYIGKEGRNEPSFVKAHDKKAFELS
KFIWGPKGIALDSNVKSNGVSKKSVAKKKIDSVKQELVFAGGSSTNGKKVEEDGGDDGCD
WFDNSSLVRMIASLGVDEYYVKQQWSLVSVESKKIVEEKYKLLQAKELEFVLEKTKFLNE
VASMFVEASKNKPLDT
>AtGeBP05
MSKKHLKPLETCFEDEEDDVIYLPPGLVTGATSKKNEEFCGGSGKVQPSEMKRRSEGTST
DMTSKRAKKVSAEDEKKVGEWTKNPYFQRLWSEEDEIVMLQGIIKFEDVTGKSPFEDRHG
FIEFVKNSISFEASVQQYIGKISQLKRKYTRKRKNGFSEGHEQKCFKLAMSIWGTKETSK
KTDLCSSPKGKKVKEEDGDVTNSDWFENSFLLPSIESLGVDSVKRKWNVVPMEFKKKIEE
RLELLEADESECKKMEEMLKVKNSECVKQKTNLLNEVIDVMT
>AtGeBP06
MKGITKKLLVSRSLKRLTAKKNRRKIHRETNKRLKPEEDPSMVKRRQDSDSDSLEKEVSV
VVTKTSDDEMVSTDTKKNYFQRIWSDDDEIVLLQGMVDFENDKGKSPYDDMTGFIDTVKN
FISFQANQHQFTTKIRRLKDKFLKKWNNGVDENSLVNAHDRKCFQLSKVIWEPTTKVEEV
GDWFVNSYLVGSIARLGVSEDVMKQSWSMVPIETKKEVEEKFKLLKDEESENLRLKTSFL
QKVNSMIIEATN
>AtGeBP07
MSHKRFNPLTASSSDEDELETPIREVEDSSSDEETDSDSDSELGKKDEVVTEVSALNNQE
SKSVKISEKSVAKRSRETHEAQASADVKKAKKVKKRGGGGGEEAETKKAYFQRVWTDDDE
IVVLEGFIDYKNDSG
>AtGeBP08
MVTPKQIDFSSCGGGDNSDDTLSHRESPRNPSSKRAAASFAAEAGEETMKKKKKKKKNL
GPPLIVRIWNEEDELSILKGLVDYRAKTGFNPKIDWDAFYSFLGSSIVAKFSKEQVLSKI
RKLKRRFHVHWEKISEGNDPKFTRSSDSEAFGFSSMIWGQGEFGNDDGMDKEMVKEHDVN
GNGAAENGTARIAQENESGEEMLKEHEETLNENGAEEIRDNDETARKAQQLESESEEEML
KEHEEPFNENGAENIRDNNGTTQIAQQSESESEEMLKEHEEVANTELVNENGAAKTTENG
TTGGKERHDDDDDDELCAVQDAFEAVMSQGLSGYQKKLQLEKLMNLGTGKRRELSDEWKA
LCVEERRLNIKKLRFSAKLAEAANDS
>AtGeBP09
MVGTKRVADSIDTNSDDTLTRNREVEVEAMLSRRKQLRTTTTRTTTTRTTPLSLSSSASK
MNWSKNDELVILGGIVDYENETKLSYRSDWDALYRYIKDCVEAKFSKIQLINKVKNMKRK
FTYNQGRSNHGEQLSFTNTDDDEIFKLSLIIWDKNESEYVSNENIDQAKDVPSGEPETND
VPCEEQDDRDVPCEEQERANIEIDNGVREKLDQAKDVPCVEQESEDVPCVEQERVSIEID
NGEKEKLDQTMDCEEQENTDVLCEEKGDKDVPCEEQENKDVPCEEQERVSIEIDNGEEEM
SSEEDGVDEVGVMEDTLDSGISFQGLGKNGVKDKSEEDDVVELGVLQEIFKEDTFFQSLG
RYQQKLLLQNLENVGVERRKELINEWKALFVDEQRLCVKKLTFAAKLANLGVSP
>AtGeBP10
MTSDHRDALFSLELESPDPDEGGVADGGESDTDEDLRDNDDVVMPGTNEAEAEDDDPEEE

DLNSPSTSSLPMVSTISATAVVSGTPTATSSTGAVTVALPAGSAVPVSVIPVDSDPKWHR
MTEIVHQRPPIDDSRRLFQRLWTDEDEIELLRGFLDYMTMHRGSSSHPPDTAPFYEQIKS
KLQLDFNKNQLVEKLRRLKKKYRNVMSKISSGKEVFFKSPHDQSTFEISRKIWNQTGKII
GFEDNNVMDFEETNNHHNTTNGNYSTFNSPSSNPTLELDSENGIEKKLTMSSSSVSRKRS
RSRIGKIEEDNKPVITPSDGPIASNVNLNEAAAVGIGGNLGGLIEETVKNCVSPVIKEMM
NGTTSMMMAAMGGGFPGLGGGGGGGGGHGFGSLSPMFTRPFNFGFGVEGGGNKAVADERW
RKQQILELEVYSRRLELVQEQIRTTLNELKTMPSGI
>AtGeBP11
MTSDHRDALFSLELESPDPDEGGVADGGESDTDEDLRDNDDVVMPGTNEAEAEDDDPEEE
DLNSPSTSSLPMVSTISATAVVSGTPTATSSTGAVTVALPAGSAVPVSVIPVDSDPKWHR
MTEIVHQRPPIDDSRRLFQRLWTDEDEIELLRGFLDYMTMHRGSSSHPPDTAPFYEQIKS
KLQLDFNKNQLVEKLRRLKKKYRNVMSKISSGKEVFFKSPHDQSTFEISRKIWNQTGKII
GFEDNNVMDFEETNNHHNTTNGNYSTFNSPSSNPTLELDSENGIEKKLTMSSSSVSRKRS
RSRIGKIEEDNKPVITPSDGPIASNVNLNEAAAVGIGGNLGGLIEETVKNCVSPVIKEMM
NGTTSMMMAAMGGGFPGLGGGGGGGGGGGFVYNLLY
>AtGeBP12
MMRLRRLLERMLRRCCFRDCSVRLTKSLCFKKLIEFDATKNQIVTKLQRLKKKFNNAVKN
ARKKGQTEDEVEYAKESEKKRFDLSIMIWGSNGVLVAGKSSKKKVAPKEMKPEETDAKVV
NEGLSIGREMVPFGSSCGLDESKLTTGWENVEDGAEKREVEEKWKKFKDKLFELLYERSV
LMNKTEAMMFKAES
>AtGeBP13
MDKANTNRSKVCGGSGEAKLTGKKRKNVSAKQSKKDAKKENSQMLKWSSKDEVLVLQGML
DFKSVTGKNPVDDINGAYEFVVHEYISTVIDEDFIEKMKSLKKKLKKKQRIYDKDPSSSE
PLYQKSSEWLKMIWGYDVESALEKPRKSKRIIKL
>AtGeBP14
MASLENPAIDSSSEFESSSEEISSSKESKPKEAPVTVPSTKTLNSPSAAVAVSDDSESEK
QSFVLTRRKKKEGAAESPAVKSGKKEGAAESPAVKSGNNEGATESPAVKSGKKRASEGTT
SRDMHVKRIKKEGDNKKGHAQRVWSEEDEISLLQAMIDFKAETGTSPWDHKDRFFDIAKK
SISFDVSKVQFFDKIRSFKRKYFGDRKVRSVESDHDKKCLGLAVSFWGSDGVSLETPVKK
KVKDESVLVKANSKEKNVKPLVKEDEQVVILGEDSEWFEESFLVPVIASLGLDEYSVKKK
WSKVSVETKKRIQEKMKLVDAKKCELLLANMDVLKEVTSVLTQTN
>AtGeBP15
MAPLESPATASSSEVESSSEEIFKSSSEESKPKDPVTVPSSKTLKSPSAAVNSKTDSSDD
SEKQSFVLTRRKKKEGAAESPAVKSGKKRAGEGSTSRDMHVKRVKKEDDNKKANPQRVWS
EEDEISLLQAVIDFKAETGTSPWDHKNAFFDIAKKSISFDVSHVQFFDKIRRLKNKYFVN
RKNKSGESNHDKKCLGLAVLIWGSDGMNVESPVKKDESILVKGKANSKEKKVEKPLVIED
EQVILGADSEWFEESFLVPVIANLGLDEYSVKKKWSKVSLETKKKIQEKMKVVDAKKCEL
LLAEMDVLKDVTSVLAQTN
>AtGeBP16
MVTPKQIDFSSCVGADNSNGTLSHRRSPRNIPSSKRAASVAEEETMKKKMKMKKKKKKLD
PPLIVRIWNEEDELSILKGLVDYRAKTGFNPKIDWDAFCSFLGSSIVERFSKDQVLSKIR
KLKRRFHVHSEKINQGNDPKFTRSSDSEAFGFSSMIWGQGDDDGMDKEHEVNGNGAAENR
TNESGEEMLKEHEEEVANTELLNENGAAKTTENGTSSGKERHDEDNDDDDELCAVQDAFE
AVMSQGLSGYQKKLQLEKLMNLGNGKRRELSDEWKALCVEETRFNIKKLRFSAKLAEAAN

DS
>AtGeBP17
MTKKLDPPTAPSSDEDDVETSEDDSSSSEEDEPIKSLPATTAAAPAKSTAVSAATPAKST
SVSAAAPSKSTAVSAAADSDSGSESETDSDSESTDPPKSGSGKTIASKKKDDPSSSTATL
ALPAVKSGAKRAASEAATTSTKRVKKDEESVKKPALFQRLWSDDDEISMLQGMIDYHADT
GKSPSADTNAFYEFQKKSISFEVSKSQFSDKVRSLRKKYRAKEGKDEPRFVKAHDKKAFE
LSKFIWGPKGIALDSNAKSNGVSKKSASKTKEKLDSVKQDLAFVGVSSTNGDDWFEKSSL
ARMIAGSGIDEYYVRQKWSSFTLETKKIVEEKFQLMQAKELEAMLDKSVRLTDLTSYFVD
ASKN
>AtGeBP18
MVSVQNLKTDQLLNFLFKNPTKFLSRFSPMAKNKTLVTPSTVKKSSDVASTSKKLSGVAS
PAKKPSGVTSPVKKPLEAVASTSSEEEEEDEPSSDSESGSESESDTEAEPMTLAAAAPSS
SNEKRQSEGKPEEERAKTETETGKKPLLFQRLWTDEDEIVFLQGMIKFAKDTGKNVSEDM
NGFFEKLKDSISFEVKTDQFVNKIRSMKRKYIENKKTTTEHDKKCYELAEIIWVSDGDAT
ALVKPKKKKLKVDEESDWFERSFVDGAFKELGPGVDEETWKKNWSLVPVKKRKRIEEKVK
SMQADELKTTWQKIDVVHEMRSLMAKYV
>AtGeBP19
MAPKQLKKIENPVVSSSEEEESASSGESATSGEESDSSADSPVKESSKKPVVVSKPSGSK
TTTKPESSTAAKRSFEKTDEMSKKKSKNSMGEEDVKKKDETLKKNLFVRLFTEEDEAILL
QGFLDFATKKENPSDHIDDFYESIKNSISFDVTKPQLVTKIGNLKKKFNGRVSKGLKKGK
NEEVMVFSKASDQNCFDLSRKIWGSNGVLYSKSKNMRQVQLGGSVKVDEDDQEPQKHRFV
ISTLSSGQELVSYLKVENPNSLGVDDTKWSAKLDKIKDGKQKRKMEKNLKKIQAKEEELS
MMRSEFVAAVTNVLSKQDNASYSCK
>AtGeBP20
MAPKKAEEVVESPPVSSEEEESGSSGEESESSAEVPKKVESSQKPESDSEGESESESSSG
PEPESEPAKTIKLKPVGTKPIPETSGSAATVPESSTAKRPLKEAAPEAIKKQKTSDTEHV
KKPITNDEVKKISSEDAKKMFQRLFSETDEIALLQGIIDFTSTKGDPYEDIDAFCIYVKK
LIDFDATKNQIVTKLQRLKKKFNNAVKNSLKKGKTEDDIEFAKDLEQKGFELSRKIWGSN
GVLVTGKSSRKKVGGTPAPKEMKLVAHSTPKKQQEEAKKPERTEAKVVNTGLSIGKEIAS
FLNADNGSSCGLDESTLTAVWAKVADGAEKREVEEKWKKLKAKQFELCLQRSGLVNETAK
MIFKAYES
>AtGeBP21
MATPTELGFSSPGGGGDDSDDNPPQKRTSKRTASETATEEETKKKKKKKTKHNTKMASPP
SNRIWNEEDELSILKGLVDFRAKTGLESKIDWDAFYCYVKGSIHVKVSKCQLMSKTRKLK
KKFLDQMEKIDQGNDPHFTRSSETEAFGYSMMIWRKIDAEYTNGVMDKAHQSESGEEVFE
EDEEVALIDKGAAKSGKSPHEAVVVVDKITTKKNGTAGKESDDDDDDVLCAVRDAFETTM
MSQGLSDYQKKLQLEKLMNLGTGKRRELSNEWKALCVEELKLNINKLRFSAKLAEAANDG
K
>AtGeBP22
MASDQRDTDFSAESPDLEEDGGGGGGGRGGGETESDEDVVIPEPNEAEDDDHDPDPDPEY
EDLNSPSMISRAPATKSSSGTVTVALPAGSAVPVASIPSDSDQKWHRMTEIVHQRPPIDD
SRRLFQRLWTDEDEIELLRGFLDYITNHRGNSSHPPDTAPFYEQIKSKLQLEFNKNQLVE
KLRRLKKKYRNVMSKFSSGKEVFFKSPHDQATFDISRKIWNQTGKIIGFEDNNVMDLEET
NHVNNANGSSGFNVSVIGNANVDVDSENGLEKKVTISRKRSRSRIGKIDEDKPVLAPCDG

VIPNAVNLNENVAVGCDFGDGRNLGVLIEETVKNCVSPMIKEMMNSTTGMMMAATGGFPG
GGAHALGVLSPMLMPSMNLGFGGKGVGDERWRRQQILELEVYSRRLELVQEQIRATVNEL
KTMPNGG
>AtGeBP23
MDKANTNRSKKEVCGGSGEAKPTGKKRKNERTLTNKNVNAKKSMMDFKALTRHNPSDDMT
GAYNFLHEYISVDVYSYEFVEKMKSLKKKLIEKMGINAKDLSSSIGSELLKLIWRYDVKS
VVEYNLLKIEQANFTSHPQISFNNSEPKKTNL
>OsGeBP01
MPCGYGVPIISTLDFTARRGTTFASHQYDTGPFYDEIRRRLSFDFTKSQLVEKLRRLKKK
YRLCAARMASSPHAAAAGFAFRTPHEGAIYDLARHIWPPALKRDGTASDDDDINPAAAAA
TAAVMTPVAMEDGFGGSAPTPTPTPRGRGGRRVRRRMAQEQEAAALPSAPALTSTDGAHQ
EPLVAAMENTLPQIAQLPPVSETEPMPVIANGANEEAVRSVLSRLLKEFITSFAVVGQTG
PGMGLNMGFGGAGLNADIAGLGFGIAGLNPGVPGADRWRQHQILELEVYLKRIELVREQV
TAALDELRSSEG*
>OsGeBP02
MRRRSPPPAAAQPPRPPTRAAESGEEGGDPGKDEVAAPPKGDSALPRSGSGFGSGNAAAP
DGPAKSPMKPKKRVTAPPSSDHGSRENLAAGSPSNEVPTPQGDDAAQEDDLPESPPKSKK
NRKKKKKALRAGDSGKVAAPDDPAEPTRPQQDVGEVDGRLAESEQEVPPNAGKAKKKASA
QARKKPSAKQHAAAQEEEDGDLMAEAEEEVAPWQGDEEDGKGPLPQRKSKRVAALSGSIC
PPDPKRAKIVDAQKPGFGRKWNGNDEIMILEALVDQIRSGGNVPQEPGHPLFHELVQRLE
GRTFNHSDVRDKVRSLKRRYNDVVLSGLAITKDHDLQLHELSCEIWGRSVAHAGDDKQRC
LARDEQSSLARDEQKSFARDEEKSLARDEQSSLARDEQKSFARDEKSLARDEEKSLASDE
QRSFDDMCKQFPLLAKEIKVLMEGQPAIMELFPRLDGDQVVAIEKKLENLRWIDMKRKKK
MAVKMAKIRKGLIYKLEGAAILADGNMIH*
>OsGeBP03
MARKRRAPTPPPPPPPPPEAESSADSSGEEEEEEEEEEEEERESPVAPPPQKPSGRGAASS
DEEEEEEEDSDTDTYAQGFQLRKVGGGDEEGEEVEGDSSESEPEPEPVKKESAKKAKAEA
KKKRAAPEPAPSGKAKKKAKPEKSSSAAAPEPAPSSGKSKKAAKAEAAKAAAAEPAPSTGK
VSKYNLAPEPSPSSKSGKALSRWTTDDEVKILEVLVAHFKSHGTQLKVEGIIAAVGDSLE
RKSIKYSDMYEKVRRLKQRYEATAKKVEHGGDLPAKEDDLRMYQLSSEIWGKNAKDAGNS
SKNKKGQAKKDKVSGDSKEAAKEDKVDEAAIAVNEKGGTLAENKKGKTNKQKTGMETKVG
SSKEAALAASPTKGKKKGSHKDKLDEEAKSGTAKVTSTIATDDDDGTLGGSKREKAGKEE
LDGDTHIVMPKEATTTAASDDGTLVGSKKGKADNGKLDGDTHSVMPKEATAGTQNGGILT
GGENHKEKVDKDANVPSIRREYAELQSLYPNLASFVNGIEAQHPCGSTFKRAFEFISDDK
ACTLESKIKKQKIAEVRMQLRLADTKKEVANALLGLLD*
>OsGeBP04
MPSKRPSQSAMDAGAAPASPSPPRSSKKRSSRPKPRAGDAARHPAPNPSPPPAAAAPASS
RSRERERKRRQRGAFADPAAVTAPAAGGQHGGAVQKLWGDADEVALLAGAAAFRARAGHV
PRLPDMGALFDSIRGSLSPHIDQAKVYYKLKRLKGKYLHAAPGASAGPHERRVRDLCASV
WGADLEPLAEGDDERAAAAAAAAAADQPRTVPDAAAMLPVLTEMLDEYWKTDGRALSSVS
LAKGLSLLGTEEARFIEGKWRRQLDSEIQTQMRRHDLAKEVYALLMDAIKALGP*
>OsGeBP05
MAPKRPTPPPPPPPVASSEETASGSDSDESEEEEEESPLAQPAPVVSNKGAESDSSGEEE
SEEEEEEDLVRSSATKSRDPPQENREEDDSSDEEEDESSESEKAEAPPPPPPNPAPKQGA

EGNGPKVSSPKRQAFHRIWSTEDEVRILEALAAHRREHGSLPQTDALIATLAGSLDNTGY
GRKELQGKVSTLKRRYESTAKKKGDLPSKGHDRRLYDLSKSVWGSEAAAAAVNGTTTAPR
EFGEMCELYPHLAEEVKLLEAAHPGLFKRDFGKLDDDKAHALDMKIKKQRIAEISVVLRR
GDLTKEVTKVLAELVE*
>OsGeBP06
MLPTVVDDPSAAAAAAVGAAASSSFPDADVYGNGDSDDIDFPSDPNHATTQAFSSAAAAA
AAAGPGGGGGAGSGSGGERRPLFQRLWTEEDEIVILRAFAEFTAQRGTAFASHQYDTDPF
YEDMRRRLQLDFSKSQLVEKLRRLKRKYRNCVDRLRATGQSFSFRSPHEQAIFEIARGIW
RPTSDKHGRDADSDDDALPDAAAAVAVPAPANGEVRSPSSTRAQQRRGRRRRTAAAAAAT
ATAADASEPPQPYPPAPAPAPVKAEDALPHFFPQGAAVTATAHVHGVDPASAAASAAANA
EGGILAPLFKEMVRAMLTAGMAPPSLEPPAPGIPMKGERWRQQRILELEVYLRRIDLLQD
QVRTALEELKSTPPATQ*
>OsGeBP07
MASDQTLPVPVSVELPPAAPNPPDPTAPLLPHADDPSAPPAAAAAARKLPVKRRSPPPRP
SSPSSSDPASSDPAAKQQPQQPPPPFKFQRIWSESDELRFLQGLLGCGAQGLVFPRDLNV
FYDRFSESMPSPYTRSQLSEKLRRLKNKFRGMSARVARGLDPARLAPHDRDVLHLCSRLW
DPANAATSPFAAAGDASSGNKRRRAAPRAGPLMPPDPSGDSNSHDYNGGISAGTPGAFGD
GHGGEEMMYLEQESGHFGYHGDVAIAADGSLDGIVKVQPETLPALPSIGDIAVHSENGNG
KAVVPRSNEHHMANAVLDIFEECLREVKANGITYGANVNGGSELARRWRAQRIDELDVLS
RRLRLLVEDAAAAGR*
>OsGeBP08
MSSSRRPPARAAATEAAADAALPLHPSSPRSKKRSSSSRRAAAGDRRPAARAPNPSLSPR
GGGGAPSRKSERRRRPRSLAMAVHGHASTSGGPGLVWNDADEVALLTAAVAFRARNGFAP
RLPDMGALFESLRGSISSHIDQAKVYYKLKRNKSKFLHAPPQATTTTPHDRRVRALSAEL
WGSELAPPAVEGDADAAEAADERDAEEGYIGGNLHVSVRLPVVSEVLGDYWRKNGRVLSG
VSLERGLALVGPEEGRMAEAKWKRQLEVETQTQGRRHDLAKEVCAMLIDAVRGLGP*
>OsGeBP09
MLPAGGYPSAAAISFPDADVDDDSDDGDFTDAPLIDPSDPTFPNPASSSAAAAAMATPAS
AGGGSGSVGGVGSSSGGGERRPLFQRLWTEEDEIVILRGFAEFTAARGTAFASHQYDTDP
FYEDMRGRLQLGFSKSQLVEKLRRLKRKYRNCVSRLRGSGSAFSFRSPHEQAIFEIARNI
WRPTNKHGRDGDSDDEDATQVTPAPVPVNTSPNDHNANYSDNGGLGTPQIAGHATSAGCF
RCRQELLDASV*
>OsGeBP10
MAPKRPAAASGSASEASDCEAGGAGRRRSPSRSRSPSRSASPSRSRSKTPPHNAAVLSST
PASAAVDFVAASDSDAGADADARLASPRRSRERSPRLHSDSDNSAAATAEAAEAAAAAAF
DDGDDEGNATPPPRSRRSSRVEATGVKPISSRPMDASRRPAAASSQSQRRSKRPRSSPTQ
HSPEQHKRPPRVWNPQDEVTILRALISYRAKNGALPGSSQDTGKLHNMIRGQLSVKASTT
QLSDKVRRLKHKYNLILTRVTKSGRDPDLPTEHDREVYELSKKVWGTKSGGAGAGSGGGG
GGGGGRVYENAEVVQSDEEQGSRDDSDEDMESGWDDRDHRNRRLKAIVVANGNGNAVTGG
RSVHGNGSGKGDVADKGKDMYPYLWEAVEELSKEHPSGTAFRKAFGVLDGSRARAMEEKL
NRFRLSEIRQQLRRMDLMKETIKMVLDALEGSDL*
>OsGeBP11
MAPTLPVVPAHGEDKKKKKKKKKPLKPSQEEEEALPLPLPPPDRKRKKASEPVNSPERAK
KKKTATPHEPPSAKQQKRPLPFQRTWSPNDEVLILEAMAAHRQEHGKVPTAAELFPVLNG

RLDRKRLTYKKLADKLRTFMRRHGRDAKNGPPTQAHDRRLYDLSRNVWVSQTQPPNLSAN
ANSNIAGGQPNQHDAMPTAGKAFDKMRDSYPNLTQALLLLVGTDLEKALTAIDETKAQAL
DLKVSNLKKELSEAVMESATIQSTESSKIPCFPSTKLQPEFGAEIEKNFQLEHLDEMKGT
QVKLARMEQEILELKQNFLAFQSQQMADSKQQHDKSSAKGIICESSESGLRSIVADNNIL
CNTLQKEMVVQQKLSCGKTKEVTSKHRHPQKLVVFPF*
>OsGeBP12
MSSWWSWRSLFSSLANANGGGSNADASSGSGTSSSPPVHEAQQAAARRRSARTKKPPEEE
AAGSQPQPKTRPSPASKASKAKVLLLLGDGEPKKKPAPNPTPTQKRSNKRKRSWSRADEL
RILEAMANHANAHGGALPEASDLFAALASSLERGDADLPKLADKVHKLKRWYDNARLPQR
CPTDDDDDTRRLFQLCGKVWGPPSTVLRTSPRQRHKVVGVLVQGNGANPQPAAALKVKEK
RVRRELSELYVLYPCLAQEVKAHANEYGELIGTAFQFIGDDEARCYDDRYRKMLVDKLNM
KKEHADVTKSLLCTLAGYIN*
>OsGeBP13
MAPKRPAEEADAAAAAAAAAGGSASEGSDAEASAEAARGHGSSPSPSKTPPPANPNPKSA
AAPPSAVAAPASAAGSDSGAASDSPRAAGNPSGPRSIEVNSDSEDSALPLASDAYADQAA
AAGAGAGADSDDGNTSPLPPPRPSRAEAAAIKPISSRPMDPPPRRSAGGSEPRAKRPRSA
AVASSAEHSKRPSRVWSQADELVILRGLITYRTKRGVLPGSTQDIGKLHSYIRGQLSAKV
STTQLSDKVRRLKQKYQMLATRAKTGKEVFPTPHDHNIYQLAKKVWGTMSTAGEGGGSGY
DNADAGESEEEQYGRESDDDMESGRDNRHRKNQRSVPVTMANGNGTGIGAVNAIVRGRSE
FEKGKDVYPYLWETVEELSSQHPTGAVFKKAFELLEGSKAQVMEEKLRKFRLTEMRQQLR
RMDLMKDTLSMVLDALEMAD*
