# Supplementary material for: Genome-Wide Characterization and Expression Analysis of GeBP Family Genes in Soybean
Source: Plants (Basel). 2022 Jul 14;11(14):1848. doi: 10.3390/plants11141848 (PMC9318833; doi:10.3390/plants11141848)
Supplement: Supplementary file 1 [file plants-11-01848-s001.zip › Supplementary file 4.pdf]

>GmGeBP1

TAATTAGCTCCTCAATTTTTTTTAAAAAATTGTAATTTAGTATTTCATCTTGTAAATTTT
TTTTAATTGATTTCCTAGATTTTTAAAACAACTACAAATTATCTGGCTTATAAACTATCT
TCAGGGACTCAACTACAAAAAATTACAAAATCAAGACCCCAATCACAACTTTTTTTTTTC
AATTTAGGAACTTAATTAAAAATTCTAAAATAGTTTAGAGACATAATGATAATTTAACAA
AATTTGCAACATTTTATTATATTTAATGTTAACTGAAGGAACTTAAATGAAAGCAAAAAA
AGTACTTTGATTAAATAACTAATTAAGATGAACAAAATATTCATTTGAAATGTTATTTTT
CGCTGAAGGGGAAAGAAAACAAGCGACAAACAAAATGCTAATTTAATTTTTTTATTGTGA
ACATGCTATTAGCATAAATTAGCTTCCGTAATAATAGATGATTAATTTTTATCAGAATAT
ATACAAAATAAATATTCTCTTTTACATATAATTTATTCTATACTTAAGAGTTAATCATGA
TTTGATTTTTAGAGTCATAATTAAAAAACACGAGTAATTATCACTTTTGTTCATTGACGT
TATTTTAATTTTAATAACAACGGAATCTTATAAAACATTACTTTTAAAATATATAAAATT
TTATCATGAATTTTTTTCATGTGAGACAAAAAGAACATAGAAGATAAGGATTTTATGTTT
CACTTTCAGATCAACTTGACCACTGCTGCAGCATAGTAAAATGTGTATATTTGATCTATA
TTCAGCTGGTAGTTGAGACCTCCAAACTAACAGATTTCATTCATTTCACGTTTGTTTTCA
ATTGACAATTACTAACTGAAGAGTGAATACGTTGACGTATTCCATTTGCATTACCAAGTC
CTGTTTTGTTTTCTTTGTCATGCTTTTTTTTTTTTCTCTTCTGAATATGAAAAAGAAAGT
GTATCATACAACATCGTGAGGGCACATTAGAAAAATTGTATTCATTTCATTTTAATTTTT
TATATTTTATTTTTAAAAAAGCAATAATTAGTCGAATAGTTTTAGAATAGCAGTCTGAAA
AATAAAAACTATTGTTTATATTTAAAATATTTTTAAAAAAATATATTCAATAATAGATTA
AACACGCTTTAATAAATATATTTAGAACATTTGCATTAAAATTATTTTATTATTAAATAA
TTTTTATGTGTCTTATATTTTTATTGTGTTGGTTAATTTTAAAAAAAATATTTTTGGAAA
TTTTATCATAAAAATAACGTGATAATAAATTGATTTACACATAACAGGAAACAAAAATTC
TTGAGTAATTTAATTAAAATATTATATTGTTTTATAAAAACATATATAGCATGTAGTACT
GTGAAACTTTGTAGGTTGTTAAATTACAAATAAAATACTAATTAAATTTTAGTCATGAGA
GAATCATTGAAAACATTGGCCTTAAAATATATTTAAAAAATTAATAAAATTTTCATTTAA
TATAAAAATAGATTCTTTATGTTAATTATTTTGTTGTGGAAACTTGGTAGAACATAGACA
TACTTTTCAGTCAATAAACAAAACACAGAGATACTTCCTATATAATTGGACAGAGTTAAT
TAATATTAAAAAATTTCACAACAAATTTGTGTCTAAATTTTGTATGGCTTCTTCTTGATA
GTTATTGAATGAGTGGATCCTACATAATACTACTTTTCAAAGAGAGATATGGACATAAAA
CTGCTATGCGTATGAGAATATCCTTTTTCCATTTTGACAACTCACTACTTTACATAGTAT
AGTGGGCTAATTCTACTGTATACCTAGAAACTTTTGGTGCTTGCCACCCATCTATATTAA
TTTTTACTATTTCAAATATAAACGCATAAGATTATGATATATTCTTCTTCATTTTTTATT
TATTATAAAGAACATGTTATATATTCTTAAATCCGATAAACTTGCCTGATTTGTTTAACT
TCCCCCACCTTATTCTCTCTA

>GmGeBP2

ACGTACATTTATAACTCTTTTTAAAAGCAATAATTGATTTGTTAACAATAGCACATGTTA
TCTTATTTTTATTAAATTGAATTATTTTTTAATATTTTACTTATTATATGTCTAAACATA
TAATTCAATTAAAAAACTTGAATATTTTATTTTCATTAAACAACAATATTATTTAATACA
TAATCATCCTATAATATACACAAATGAACTGTTTATCCCGTGCATTGCACATGTTGTTGG
AATTTTCATTTTTTCCTATCAAATCCAAATTTAAAAATGAAACATTTTAATAAAAAGATA
TTAAAATTCAAAGGTAATTTCAATTGCAAAACATTAAAAATATACCTTTTTAGGAGTTAA
TTTTGTTGTAAATCTCTTAAATTAATTCATGAATTTTTCTCTCAAAGAAGGTGTCATCAT
ATATAACATTAATGTGCACACAATTCAGGGTACAAAGCAATTTTGAACAATAAAAAAAAC

CTTACATACCTTGTAGTGGTGATGATGAAGAAGAAAAGTAATAGAGTAGATAAATAATAC
ATCAATCATCTTGTACCATCTTGTTTAAATAAGAAAAAAGTTGTATCTCCTTCAAATCCA
TTAAACCTAAAAAATCATGAGCCACCTTCTGATTTTAATTTAATCTTAACGTTATTATTT
CCTAAACAAACAAAATTTTTACATTTAATTAGTTAATTCATTTTTTTTCTTATCACAAAC
ACCTAAATATGAATGGAAAATAAAATAAAAGAATAAAATGAATTTTTAATGGATGATTGA
TGATTTTATACCTTCTACGATTTTATGATTATCCATTTATTATTTCGATAACTTTTTCCT
TTTTAAAATCATGATAAATGGGATTTATTATTTAGAAGCATAATTTAATTTATGTACCAT
GTTAAATAAGACATTTTTAATGTAAAATAAAGACAAATCTATTTCAATAGAAATTTGTTG
GAGTATAAGTGTGAGGTGAAGTCCTACCTCAAGTAGAACTAGGAAAAGTTGAGCACCATA
TAAGTAACGAGAAGATCCATAAACCTGAGCCTTAAGGTTTTGGGTTATAATGTGGTGTCC
CTTATATAGTTGCTCATGGTTCATTGGTGTAAATCTACATGTTACTCCCCTCAATTCTCC
AACAAAATTAATATTAAGTATATAAAATGATAGTTACAAAGTTTACAATAATTATAATTT
ATGAAAGATTAACATTAATTGTCTATTCAATATCATTTGAAAATAAAAGATATATTTGGG
AAATAATTATAGGTATTTAAAATTTAAAATAAAACATAAATTACTCTCGGAACCAATTTA
AGATCATATTGAAGAAAGTATATTAAATTTTCATTTTTTACCTATAATTTAATTTATTTT
TTCCACTTAATTCTTATACATAGAAAATGATATTCAATATTTTTAATGGAACATAAAATC
ATCATCATTTCTATTAACTATAAGTAATATAGTATTCTATAAATTATATTCAAAAGAAGT
TATTTTATTGCACTCATATAACTATATGAATTTAATTTTATGCATTATAATTGTATGGTT
ATGTATAGAAAATGAAGAAAAGCTCTCGAGAGTTCTTGCACATTGTCATTGTTGAAATGA
AAGAGTCTAAAAAATCTAAATAAAGAGGTACCAGAAAAAGAAAGTGAGATAGGCAAGAAA
AGTAATCATATGCCCAAATGAAATTTGAATATGGTGACAATCATACCATTGATTAGATTG
ATTGACCTTTCTCTATCAAGCACCATAATACCTTGGAGGAATTACATTGGAATGAAAGAA
ATTCAGAGACTGTGCAAGCATGAGATAGGTATAATTGATGATGACTTAGAAGAATTAATT
AATACTATTTATACCAATAAGATGCATCTACTTTTTGATAGATACATGAAGTAGTTATGA
GATGGATAACTTTCTAAGAAGTTTTTAAAAAATGTATTAGTGAAGAAAAAATCACTAAAC
AATCTGGTGTTATTTGTTGGG
>GmGeBP3
TGGGAGTGAGTCGGTTAGGCAAAGGGTCTAATGGGTGCTTTGCTTTCACGGGTTTGTTGG
TGTAACTTTTCACTCACTCGGTTGCAATAAGAATGAGAATGAGAATCTGGGAGGTCGTTT
ATTAGTTAGTGGACCAACCCAGTTGCCCCACATGATTTTCTTTATTGCTTAATTTAATAA
GTTTTTGTCAACTTTTTATCAAATTATTATTATTGACTCAAATGTTGATTTAATATTTTT
ATGAATCTAATTACTATAATATATTCATATGACAAAGATTTTAAATTAACATTTACCTAA
TGATTGTTATATATATTGTTATTTTTTATATTATTAATTACTTTAAAAATCATGTAACTG
ATTTATAGTATAATTTTTTACAGTCATAATGTACCAAAATTAAATTACACTTTAATTTGA
TTTGTCTTCTAAATATTTTTATTATTTTTTATCCTTGTTAAAAAAATTAAATAATTTTTA
TCATCACTTAACAATCAAGTTTCGTGTATGTTGTCACAAAATAATTGTTTGGAATGATGT
CATTATATAATAAAATCAATTAAAAATATATTTATAAGACTAGAAACATGATTAAGTTTT
TAAATTATTTGATTGAATCTCTCACAAAATATTGTAACCGATTAAATATATAGTAAAATT
GATGTTAAAATTTAAAAATAAATATAAAGCTAAGATTATGGAAAAAAGAACTTATAATTT
ATCAACAATAATATATTTTTATATAATAAATTAAAATAGAGAAAAAATATTATACATTGA
TAATGTAAAAAAAATTACACAGAAATCTCATCACAATTTAACATATATGGTAAATTTGTT
AACTTTTAAAATAATTATTTTAATTATTTTTCATTCTTAGTAAAGCTAAGAAAAAAAATT
ATTTTTTAATTTCTGTTTTACTTCTTTCTTCCCCGTAAAGATATTAAATGGAATAAACTA
TTTTTTCATTGAAATTTAAAAAATGAACATTTAAATATCCTTCAAAAACAAATAAATAGT

TCCAAAACATATAAAATGTAGTTCACCTTGTTATACAACAAACTATTATTGCAAAAATGA
GTGAATACAACTATCATTAAGAATTTCATCTTTAGACAAAAAGTGGAATACCACAAGGGT
ATCCTTTGTAATTGATACATGTTTTGTTAAACTGTCCATAAGAACCATATTCTCTTTAAT
CTCATAATTAGATGTTCTCAATTATTCACAAAAAAAGGCATTCTCAATTCCAATATTCTT
TTTTCTTTGCATATGCATTTCATATATTATCATCACAATTATTCAAAAAAAAAAAAATATC
TCTCAAATATCTCAAACTATCATTTTTTTTATTTGTATGATATTTTTTAGAGAAAAGGAA
TTGTATACTTGTAAATATAATCATCCAATTATAACCTATTATAACCTATTATGATAAATT
TATTGACTTTTAAAATAATTATTTTAAAAATGTTTTACATCGATGTTTTAACCTAAAATG
TTTTAATCTTAGATACACATTAACTTTTTGGACCCATCTAAAAATTGGTCTTCCTTATAC
TCTCTTATAGACACTACTATTGGGTTGACATTCATAAAATGATAAGAGCTTACTTGAGTC
CAAATTGAAGAATCTTACACCATAGAAGTTTACACAAGCAAATTAAAGTCAGTTTTTATT
GTAATGTATTTTAAGCTTGTTAGAAAATATAGTTAGATGAACAACAAATTTTCTCTGATT
TTGCTGTTAATGTCATCGAATAAGTATATGATTGTGATTGTCCTAGCTAAAAGTTTGTTA
GGAATGCTTTGAAATTTCAACTAGTAATGAACGTTAGTTTGGGAGTTATTTGAAATCCTT
GGCAATCCAATTTATTTATTTTCCAATTACTACTTGTGCATGCCATTCTTTAGAGAACTA
CCACACCAATATAACAGCATAGATTCCATTTGAGAACTCCTTATTTGAAAGCAGCAGTGG
GTTCTAGCTAGGATATTCCAC
>GmGeBP4
TTTGTTAATTGTTAGAGTTTGAGTTAACAATTCACCAAAGATAACAAATTTCTCATATCA
TTTTCAAGGACTTGCTTTTCATCTTTGCTGCTAAATTTCAGGAATATGAAATTTATTGTT
GTTGGTTTTGAAATTTATAAAAAAATATATATAATTGCACATGTTTTATACTTTATTGAA
TGAATTTTGCTTACAGTTTTATATTTTTAAATAAATTTTAACTAATAATAATATATTAAA
ATACTTTATATCTCAATAAAAACAGTTTTTTGAAACAACTGGTTAGTTGTAACCTCGGAT
CTAAATTTATGAGAGTGTTTGGGAATATCCAGCAGAATGTACTACCAAATCCCAATTCAT
TTACGATAAATAAAATAGTGATTCCACACCATGATGTTTTGTCTCACCATCTATCACATA
ATTAGGGCTACAACATCAAAATAAAAAGAATTATATTACATATAAGAACTTTATAAAATA
CATTATACAATTCTATTTTTTTCATTATCTCTTGCTTCACATCAATTATATATTATTGAT
AACATGATTTTTCTACATGTAACTTTATATTCACGGATAAACATGCACCCCATTTGAAGG
TATATATAGGAAGAAAAAGAGAGAGATGAAAAAAAAAGATAGAAAATAATAATAAATATG
ATAAATGGCGGAAAGTTTATAGAAATACAATTGAGTAACACATGTTTTTTTTTTTAGGCA
TCATGCATATTAGTTAATTAACAACTAACATATATGGGAGGGGGGGGGGGGGGGGAAATG
ATAAATCCTCCGAGACAAAGAGACCAAATAACTGCCGAACCATTCAAAAGCTTACACCTG
TCAACTATTCACTTAATTTTTTTTTTAAAAAATTCCTTCCAAATTGATTCCTTGTTTCAC
ATCTCTCTTTTTTTTAAGATCCATGTCATCTCTTTCTAAATTTTCAGTCCAAAACCCTTA
TTCATGGATGAATCTCTTGGGATTATTCAGAGCAAAGGAATAAAGAATCTCACCCACAAA
AAGTTTATTTTCGTTCACAATCTCAAATAAAAAATGGGTTGTTGTTTAGATAGCCTTTGG
GAAGAGCACACTTCCTCGTGTTGTTTACGAGAATCACAAACCTCGTTACCAACCTGAGGC
ACGAAGGTTCTGGAGTTCGCCATTAAAATGTTTATGCCATCTAAGTGGAAAGCTTGAATG
TTATATGTTCTTTGTATAGTTTTGGGTTTACGCCATCTAATTAATGTTCTTTACTTGCCA
AGATCCACCAGCTATTGTAACTTTTCTAATAATCCAACGTTGACACTTTAAATGAATAGG
TACAAATCACTAAGAATGCCAAATACAAATTCAATGAATAAATAAAACCAAAGTGTGTTA
GAGTACACAAATGTCAGTTTCTACATTTGTAGGCTTTGAATTCCCCAGTTTTGAAAGTGC
TCATCCTGAAATGTTGGGTCGGTGGGGGAGGGTGGAAAGGTGGGATAGGGTGGTGGGTGA
TTGTGTTGTTGAAATTCGTGGGACCGAAGGAGGAGAAGCTGTGTACAAGGGAGTTGTGGA

GGGAAGTGAAAATCATGTATGATTTGTTTAGTTGTACGTGAGTGATGAGGAAAGAAAACG
AGAGAATGAAAGAGAATAAATGAACAAAAGAGAAATAAGTAAGAAATACGGTATCTTGAG
TCATCTTTCATTTTTTTTTCTTTTCAGGATATTTATGTGATTCATTCATTGTGCAGGTGC
TTTCTCTAACCATAGTGCTCTATGACTATGAGACAATAAAAATAAAATAAATGCTGACAG
GTGTCAACCTTATGTAGTTCGATAGAGTAGTCGACTGATTTTGTTCGGTGGATATAGAAG
TCTTTGTATATATATATTTTCTTAAAAAGTCTAACTCTCATAATTTTCATCCGAATTTGG
ATCATATGATATGAATTTATAACGATATATTTTTCCCTTATTATTGTGGAGGTAATAGAC
ATGCATTCTATTGATAGCAAA
>GmGeBP5
GAGAGGCTAACGGTCCAGCTCCTCTAATCACAAATTCACAGGTTGTCAAATTTAATTAAG
TTAAGAATTAATCTATTTGATAATTTTATTCCCTTCGGCATTTCAATTATATTTTCAAAA
AATATTTAATTATTAAATTCATTCACCCTCGAGTTATCTTGTAAATTAGTATTTTAACAT
GATAAAAATATAATTCACATAATAAAAAAATATTTTTAAATATGTGAATTATATCATAAT
ACAAATCTGTAATAATAAATAAAAAGATAAAAATAAAAGATGTTTTTAGTTATTGATAAT
TTCTTTTAAAAAAATTATATTTGATTTTTAAAAAAAGATAAAAATAAATTGAAAGATAGA
AACTGTTAAAAAAATCACGTACGTGCATAAAGATAAAAGAATAGTGGTTTGAGCTCAAGA
TGGTTCGTAAAGAAAATTTGAGATAAGTATTCATTAAATTTACAAATAAAAAGATAAAAA
TAGATTGAAAGGTAAAAACTGTTAAAAAAAATCAAGCATATATGCATAAAGATAAAAGAA
TAGTGGTTTGAGCTTGAGATGGTTCGTAAAGAAAATTTTAGATAAGTATTCAATCAAATC
AAATTAGAGAAATATATTTGAAAGAGGCAAATGATAAAAAAAAAAAAAATTGCTCAAGATT
ATGATACTAAGATAATACACGTGACTCGATAAGAATGATAATAACTTTTAAAAATATTAT
TGTTTAAGGAAAAAAAAATCTAAAAATATTTTCATCTAAGAAAATCTTTAAAAGTAATCG
TACTCATAATGAGAATTCGAATTTGCAACATGAGAGTACATTGTAAAAACAAATCTTTGT
TTTTAAATAATTAAAACTTAAATTTAGATGCAAGCTAAGGTGATTCAATACAAGATATGA
TTTATGGTCAACAAGTTTATAATGACCGGTAAATTTATCAATTATCAAGACTTAATAAAA
TATTTAGAGCATTCCAAATGTAAATTTATTCTAAAGATCTCAAAGTTAGGATATGTGTCA
TATCAATTTTTAAGAATTTTACTTACTTTTTATTCAAATAATAATCTCATTAAGAATTTG
ACTTGGATTCCACGTGCTATTTCACATATTTTTACCATGTGTGCTCTTCTTTTTATTTTT
AAAATTTCAATCTTTTTTTTTTTTCAATTTGCAAGCTTTTTACTTTACGGGGGGCAAAAT
TGAAACCCAACAGATTAAATGTCCTTTCTCTCAAACTACTCGTCTCTCTGAAAAATTCTC
AACCATGATGTCGTGGTTCCCCCCATCACACCGTCGCAACCACCACCCGCCATTCCTCTC
TGGTTTCACACTTTCTAATTCTGCATCTCACGCTTCCTTTCTTTCAAACTCTATGAAAGC
TATAGGATTGAAATCGATGGAGAACGGATGTCTCTCTTTGCCGGATGCATGCACCCAAAG
GACGACTCTGTCTCTTTCCTGCACATCATTATAGCCCCTCGAACCGGTTGGTCATCGAGG
GTCTCCTCGCAACAACATAGTGCTGGCATGTTAATGGTGAATTTGATTTTGAGTTAAATT
ATTCATTTGATTTCTATAATTTCATGATTCTTATTTTTTTTAATCTTATAGTTTAAAAAT
AGACTTTTTAGTCTATATAATTTACACTTTAATCCTTTTTTAGTGCATGTAGTTTTGAAA
ATGATCTTTTTAGTCCGTTTCATGAGTCTTACTTTTTTAGTTTTTATAGTTTTCAAATTA
TAAATACTAAAATAGAATCAAAATGTAAATGATAGGGACTAAAAAGATCACTTTCAAATC
ATAAAGACTAAAAAGATAAGAACCATAAAATTATAGGGACTAGATGAGTAATTTAACCTT
TGATTATGCTTTGATTTTGAAATATTCCAAAAAGCTTGAAATGTGTTTTTTAAAATAAAT
ATAAGAATTGTTTTTTCAACAATAAAATAAATATACAATAATGTTTATATTTTCTTTAAT
TTATATACAATAGAAATATAG
>GmGeBP6

ATTATAAAATTTATAATTTTTTCTTGTAATTTCAAGTACATATTCAAGGGAATTAAATCA
AATCAATATAATAAAATCACATATCAAATTATATAGATAAGATTCTTATAAATGAAATAT
ACATTAAAATTTCCTTTTTATGTGTCAAATGGGATCAACTTGATTTTACCCACCAGAAAT
TCTCAAAGAATAAATTAATAAAAATCAACTCACTTTTTTTCTGTAATATTATAAATAATC
AATCTAACTCAACCTATCATAAATTCATAGACTAAATAAGTTTACCTACCTAAAATAAAA
GATAAAAAAATACTGTAATTTTTATTTACAATACTTTTTTTAATAGACAAAACTAACTTTT
TCTAAAGGTAGAAAACAGCAAAAAAGAAAAATTATTATATTACTACTACAAGGTAAGCCA
CGTTACACGGTGTCAATAGAATATAATCGTCATTAAAAGAATGCAATTTATGTTAGTACG
GATGGTTCAATTTTGATTTTGTGATAAGTTTGTGAGATGGAAAAATACAATTATTCAATC
ATAAAACATTATGTATTTATTAATTTTTAAAATAATTATCTTAAAATAATTTAAATTATA
TTTACACAATAATGTAAACTTGTTTTACATTATAATTACATAACGAGTAAACTTAAAAAA
TAATTCTTTACTTTAAAATCATAACTTGCAAACAACACCTCAATCTCACATTCAATTATC
AAAATTAAAATCATCAAAAGAGATAAATGTTTTATATATATTCATATTAACCTTCATTCC
AAACTAAATACTGGAAAAAGCAACTGAAAACTACAAAGTTATAATTAATTCCTCTCACTC
TCTTAGGAGTTGTATTTGGAGAGAAGAAATTAAAGTAAAAAAATAAAGGTATGAGACCCA
CCTAAAAAATTTAATTTTTTTTTATCCTTTTCTCCTTATCTGATCTCAAACCAAACCAAGA
CTTAGTTTAGGCAGCATGTGTTGGTGTTGAGTTGATAAATGAAGGAACGGTTTTTTTCCC
TCTTTAGTATATGCAAAGCTCTAACTCATGAAATAATTTGATGCATCATAGGATTCAATT
CGGTGACCTGAGTTTTATCGATTCAAACCTGTAATGGGTCGAGTTGGACAAGAATGTAAA
TATTGTTGTCAATAAGATCTCAATTGCAGAAGAATTAATGATGGCTTCTTAACCACCATA
AAATAACCATGAATGACGACTTTGTGAACATAGTCACAACTTCATTTGTAAAGAAACTTG
TGACCGAGAAGCCATTAGTGGTGACTTCAAGATAATTGGTAAATCCAATAATCGTTATAT
CATCTCGTCTACGCCATTAAATATTTCAGTAAGACTTGATAATTGATAAGTCCACTGATC
ATTATAAACTTCTCCACCATAACCCACAGCTTGTATTGAAGCACCTTAGCTTGCATTAAA
ATTTAAATTTAAATTATTTAAAAATAAAGTTAAAGATTTGTTTTTTACAATGTACTCTTA
TGTTGCAAATTCGAATTCTCATGTTGAGTAAGAATATTTTTAATGATTTTTTTATAGATT
TTTTTCCTTAAACAATAATGTTTTTAAAAGCTACTATCATTCTTATCGAGTCACGTGTAT
TACCTTAATATCATGCTTTGAGGGAAAAAATTATTGTTTGCTTCTTTCAAATATATTTTT
CTAATTTAATTTGATTGAATACTTATTTCAAATTTTTCTTTAGGAACCATCTTGAGATCA
AACCACTATTCTTTTATCTTTATGCATATACTTGATTTTTTTTTTAAGCGCTTATATATA
TCTTCCAATCTATTTTTGTCTTTTTATTTGTAAATCAAATATCACAATTTTTTAAAAGAA
ATTATCAATAAGTAAATCAAATAAATTTAATAATTAAATATTTTTTGAAGATATAATTGA
AATTTTGAAGGGGATAAAATAAAATTATCAACTTTTTTTTATCAATTATCTTCTTAATTT
GTGATTAGGGGAGCTGAACCG
>GmGeBP7
AACCAAACCCCACGTATTAATTTTCTATTCCCTATTTTAATTTTTCAAGGAATGTTCCCT
GTTCGTTTGCGACAAACGCAACGTTGCAGTATATGTTTTATTTTTTTAATCGTATTAAAA
ACTATCCTCTACATTTTCAAATACTTAATAATTTTTTTTCATTGATACAGATATTTTTAT
TATTAAAATTTAAAAAATCAGCTGATAGAAAAAATTAGTGTTTACAATTTGTAAGTTAAA
TTAATCCAGTAATTTCCAAAAGTATTTAGAAATTTTTAATTAGGTCCTCAAATTAAATAA
AAACTACAATTGAGTATTTTATCTTTAATTTTTTTAATTGATCTCCTAGATTTTTTAAAA
CCACTACAAATGATCATTTTCTGGCTATAAAAACTATCTTCAGGGGCTCAACTACAATAA
ATTTACAAAATCAAGACCCTAATTATAGTTTTTTTAATTAAGGAACCTAATAAAATATTC
TAAAATTGTTTAGAGACATACTGATTAATTTAACATAATTTGTAATATTTTTCACTATAT

TTATTGTTAACTGAATGAAGTTAAATATTAATTAAAAAGTTAATGAAAGTAAAAAAAAAA
AGGTACTTCGATTATATAACTAATTAAGAAGAACAAAATATTCATTTGAAATGTACAAAA
AATGAGAAAAATATCTCGATCTTAATAATGAGAAATTTTATTTTATAAAAATATATTTTT
TAACATTTCAAGAAACTTTTAGAAAAACAATCTGTGTTTGGAATTAAATTTAAATAAATA
AAATGTTATTAAAAAATATTGACTATTTTAAAAATTCTTGAGTATTCTAATAAAAATATT
ATATTGTTTTATAAAAACATATATAGCATGTAATACTGTGAAATTTTGTAGGTTGTTAAA
TCACAAATAAAATACTAATTAAATTTTAATCATGAGAGAATCATTGAAAACTTTGGCTTT
AAAATATATAAAAATAATAATAAAATTTTCATTCAATACAAAAATATAATCTTTATGCTA
ATTATTTTTTTGTGAAATTTTGGTAGAACAAAGAGATACTTCCGATATAATTGGACAGAG
ATAATTAATATTAAATTTGATCACAACAAATCATAGGAAATAATCTTAAAAAACCTATAC
AACATAGCAATTATATAAAAAAAAAAAAAACTTGTCACAATTTATAATATCATACACACAT
AACACAAATTATATCATACAAATTTCAGGATGGGTTTTAAAACCAAATTTAGAACTTAAT
GGACTCCCATACGTGCTGAACTGATGAGTTAAATGGGCCGCAATAAGAAATGCAAATGGG
ATTCGTTGTTTAAGAAAACATCCAAGAGAGGAATGTCAAGTTAGAAAGTTTCGCAAATAA
CTATGGCAGACGCTTCGCAAATGAATCGAGGAAGGTCAAGTTAGAAAGTTTCGCAAATAA
ATAGGGAACTGAAAGAGACAGGCATGATAGGAAACAAGTGAGAAGAAGGATATTGATTGT
TCGACGCAAGAGAGCTGAGAACAGAAAACATTGGAGCCTAGTTTCACCACCAAGTGGATT
AGTGGAAGGAACGTGCAAGCACGTGAGAGTTAAAATCTAAAAGTGAGTCGTAATCAAGTG
AGACCAACACAATCAATATCGTTTTGCTTTTAAATGACTGTAGAGTTAAACTTTCCAACT
CCGACATGAACAACACCCAATTTTGTCCGAGTTCACTAATAAACAATACCGCAACGATTC
AACTTAATTTGCAGGTGCGAACTAGTACAGTCAAACGAGGTTATTAAATTCCTATTTTTA
CAATGGGATTTGACAAACTAACGCCTACATAAATCAGCCATTACTAAAAGAGCCAAGCTT
CAATACATTTTTATTTTATTTTAATCCTGAACTACCACCAAACCATACATAGGCTGTTGA
TTTTGACTCCAACCGAAAAAACAACATAATCTGACGATGATGAGAAGCAACGATAGAGAC
TGGAGAATTTATGATAAATTA
>GmGeBP8
CCTTTTTCCGCGTATTTGAGTATCAAGCTAGCCGGTTGTTTTGCATTTACTATCAATAGA
ATGCATGCTTATTACCTCCACAATATCTTGTTACAAATTCACATCTGCATAATCCTAATT
TGGATTAAAATTACGAGTTAAACTTGTTTTCTTATTTTTATTTTTTTAAATATATATTTG
TTAGTTGTTAACTAACGCAGTCATCATGCCTAAAAATAAAACTTATGTTACTCAATTATA
TTTTTATAAACTTTCTACTATTTATCATATTTGCTATTTTCTATCTTTTCCTTTTATCTC
TCTCTTTTCTCTACCTATATATATCTCTACCTATATATACCTTAAAATGGAATGTATGTT
TATCCAAGTTCAATCAAAAGTAATATATGAGAAGTGTAGAAAAATCATGTTATCGATAAT
GTATAGTAATTGATGTGATGGAAGAGATAATCGAAAAAGAAAACGGAATTGAAAAATGTA
TTTTATAAATTTTATTATATCTAATTAAATTCTTTTTTATTTTAATGTTGTTGCCTTAAT
GATGTGATAGATGGTGAGAGGAAACACCATATAATGTGAAATCACTAATTTATTTATCCT
AAATGAATTGGAATTTGATTTGGTATTATACTCTATTGGGTATTCACAAATACTCTCTTA
GGTTACAACTTACAATATAATTTGTTTTAAAGATCTGTTTTTATTGAGTAAAAACAAGTC
TTTTAATATATTATTATTAGTTAAAATTTATTTAAAAATATAAAACTATAAGCATAGTTC
ATTCAATAAAGTATAAAACATGTGCAATTATGCATTTCTAATAAATTTAATATTACCTTA
AAAAATAATAAATTTCATTTTCCTGAAAATTGATAGTAAAGATGAAAAGCAAGCCATTGA
AAAAGATGAGATTTGTTATCTTTGGTGAATTGTTCAGTCAAACTCCAACAATTAACAAAT
CCGGTCACATTGGTTTCCTAAGTCCTATCTTTCAAATGAAAAAAAAAATATTAAGATAGAA
AAATCTGAATATGTAGTCTTCAACCGTTGGATCGAAAAATAACATATTAACGATGTGAAT

GTACGGAAGGAACAAATATGCACTGAACTAAGTAAGTGTCCTCTTCTCAGAGAGGAGGAG
TTGTCTAAACCTAAAGGATTAGTTCCTCGAGTTTAGTTAACTCAACAGCTTAACACAAAT
AATGCGCCAGCTTTCCTCGACTATAGCGCGAGTAGAGTCTAAGTGTTTATAGCCGTAGCG
ATATAAACAAGAATACTCATCAGAGCCGTAGAAACATGTACATATAGAATACGAGAATGT
AACTAGCCCCAAGTTACAGCAGGCTCTTTCTATTATGAAATGTTTCATCTGTATCTATTT
ATTGGTTAGTTTAGTATACAGGATCTCTACCTTCTTTGATTTTGTGTTATTCGATCTTTC
GGTGTACCCCGTACTTCCTAGAAGTAGTCATTCATTCATCTGGTGGATCCAACTCCATTG
TGGGTGTGGTTTCACACGAAGGTTCGAGCTCAGGTTGGACCTCTTTCGACCATCATTCAT
TTGTGATCAAGAGGACTATACTATTGATAGATGAGTGATTTGCATTACATCGATGAAATG
CAATGCACTCAAGTAACTTTTGAATGATGGTCTTGTTTGAGTCAGGATACTGACTTTTGA
CTAAACATTAAGTTAGAGTCCAAAAATAACTAGACTTAAGTTGGTAAAATCAAACTCACG
TCATATCCAAAGTATATTTTTTTTAATGTTCATTAGAAATTTGTTAATAAAATACACAAA
TATATACTATCAATTCAAATATGTGAACATCAATTGAATAAGTTCTTTTACCTTTAATGA
TGTTGATTTTGAGTTTTAAAAATATAGGTACATTATAAATCGATAAAAGTAGACATTGTT
ATCTTTAACTATTTTGTTAAATTCAAGTGTGAAAAATGTGAGTTGAATACTATAATTTAT
TAATATTTGTGTAATGATAAA

>GmGeBP9

ATATTTAATCATGTTTTAAGTTTTTTTATTTCTCCCTAAATATATATTTTACACAAATTA
AACTCAAATTTTTCCTCATAAATTCAATGTATATTGACAATTGAGTTATACCCATTAAAC
AAAAAATCATTTATCATATAGCTCAAATCCTGCAACAAATGAGCAAGTAAACTACTAGTC
TTATTAGTTATGAAATTGTTTAAGATAAATATTTATTTTATATAATACTTAAGATTTATA
ATAAATAAATATTTTTGAAAATATGTTATATTTAAAATTTATAATAAATAACTTAAACTA
TAATATAAGGTTATTTTGAATTATTAAAATTCAAAAAGTAAGTTAAATTCACTTTAGAGA
TATAAATAAAAGAGATATATAGTTAAAAAGATCAACTATACCATAAAAGAAAATATAAGA
ATGTCTAATTGTGAGTTAAAGAAAACAAGAGTCTCACATCATTCTTATTTGATAGATGCA
GACGTGCAAATATTTTCTTTTAAATTTTAATAACTTAAGAGATGTATTTTATAAAGAGAT
AAATAAATAATGAATCATGTTGTTCTGTATTAGCGAGAGTTAAGATTATATGATTGTAGT
CATATTTTTTATGATATATTTATATTTTTCTTTGCATATCATTCAAAATTTGACTATGAA
CATATAATCTTAACTCTCTTACTAAGGTATGAACCACATGATTTGTTTGTCTCCTTTAAA
ATTCACCTATAAGTTATTAAAATTAAAGTTTGAAAGAAGATGTTTACCTATTGAGAGTAT
AAAAAGTTTTATGCAATTACAATTGTCACTACAAAATTATTTTATAATTGTTAGTACATA
AATATTAAATTATTGAGACAGTTGAAAAATAAAACTTTTGAATGACTGTGATGATAATAT
ATGAATATGAAAAAGTTATATATTGACAGTGTAAAATTTTTTACAAATCCATTCAATTAC
AATTTATTATTATGTATGATAAATTTATTGATTTTTAAAATAATTTTTTTAAAGTAATTC
AAACAGTAATAATTTATGATTAGATATAATTGTAAAACTATTAGTGAATAGGCATTATTA
AACTTATGTATAAAATGCATATGCAAAGAATATATATATATTCAAGTATATGGAAAGAAT
AGACATTGAGAATTTCTAATTATGAGAATAAAGAGAATCAAAATCTCATTCTACAATCTG
TTTATTTTAATTTATTATATAAGAAAATATTATTGTTAATAAATCATAAACTATTTTTTC
CATAATCTTATCTGTATTTATTTTTAAATTTTAACATCAATTTTACTATATATTTGATTG
GTTTCACAATATTTTGTGCGAGATTGGATCAAATAATTTAAAACTTAATCATATATTTAG
TCTTATAATAATAATTATATTTTTAGTTGATTTTATTATATCAATAACATATCATATATA
TAATCACATCATTTCATCTCAAGTTCAAGTTTTATATATATAATTATATTATTAAAGAAA
GAATTTATAATAATTTTATCTTAGTCAAACATAATTATCTTCCTTAAAAAATATCTATCA
TCTTCAAAAAAAAAATATAATCTCATGATTTAAAAAAATATCTTAGTCAAGGATAAAAAA

ATAATAAAAATATTTAGAAGACTAAATCAAATAAAAGTATAATTTAATTTTGATATATTA
TGAGCGTAAAAAAAATTATACTATAAATTAGTTATATGGTTTTAAAAAAATAAGTTTTTT
TATTACAAAATTACAATTTTATCATCTTATATAACGATAAAAAAGTAAATGATAATGTAA
AATCTTGACCATATCAATATATTACAGTAATTAGATTCATAAAAATATATTTATAAATAT
TAAATCCACATTTGAGTCAATAATAATAATTTAATAAAAAAAGTTGACAAAAGCTTATTA
AATTAAGCAATAAACAAAATCATGTGGGGCAACTTGGTTGGTCCACTACCTAATAAACCA
CCTCCCGGATTCTCATTCTCA
